# Anions of α-Amino Acids as (O,N)-Donor Ligands in Si-, Ge- and Sn-Coordination Chemistry

**DOI:** 10.3390/molecules30040834

**Published:** 2025-02-11

**Authors:** Anne Seidel, Jörg Wagler

**Affiliations:** Institut für Anorganische Chemie, TU Bergakademie Freiberg, D-09596 Freiberg, Germany; anne.seidel@chemie.tu-freiberg.de

**Keywords:** amine acetic acid, chelate, chiral pool, complexation, hypercoordination, peptide, tetrel

## Abstract

This review gives an overview of Si-, Ge- and Sn-compounds with (O,N)-bi- and -oligodentate ligands, which have the α-amino carboxylic acid motif N–C(*R*,*R*′)–C(=O)O in common (*R*,*R*′ = H or hydrocarbyl). While the amino acids themselves are encountered as mono- and di-anionic ligands, modifications at the N-terminus (e.g., extension of the ligand backbone by, e.g., additional alkane carboxylic acid groups) give rise to a wealth of ligands, which bear the α-amino carboxylic acid motif. With particular interest in the coordination features of these ligands, crystallographically characterized complexes are the focus of this review.

## 1. Introduction

**Figure 1:**
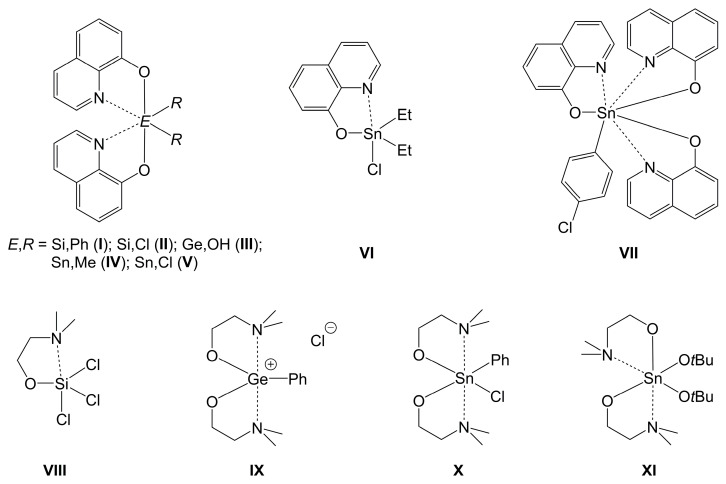
Hypercoordinate Si-, Ge- and Sn-complexes **I** [26], **II** [27], **III** [28], **IV** [29], **V** [30], **VI** [31], **VII** [32], **VIII** [33], **IX** [34], **X** [35] and **XI** [36] with mono-anionic (O,N)-bidentate chelating ligands.

Coordination chemistry of the heavier tetrels Si, Ge and Sn has attracted researchers’ interest for decades. Simple complexes of tetravalent tetrels with a higher coordination number were reported more than one century ago, e.g., hexafluorosilicate ([SiF_6_]^2–^) [1], a related ammonia adduct ([SiF_4_(NH_3_)_2_]) [2], hexachlorostannate ([SnCl_6_]^2–^) [3] and the Ge-analog ([GeCl_6_]^2–^) [4]. (The somewhat later dates of reports of various Ge compounds can be attributed to the discovery of the element germanium late, in 1886 [5].) In further studies, chelating ligands have given rise to a wealth of compounds in which these tetrels exhibit coordination numbers five, six or even higher. Thus, coordination chemistry of these tetrels has been thoroughly reviewed over the past decades; for examples, see [6,7,8,9,10,11,12,13,14,15,16,17,18,19,20,21,22,23,24,25]. As these tetrels (when tetravalent) are oxidophilic, which is manifest in the natural abundance of minerals such as cassiterite (SnO_2_) and quartz (SiO_2_), chelators with anionic O-anchor groups have proven successful in binding additional lone-pair donor sites (e.g., amine or imine N atoms) to the tetrel atoms, thus enhancing their coordination numbers. Figure 1 shows a selection of tetrel complexes **I**–**XI** [26,27,28,29,30,31,32,33,34,35,36] with two simple bidentate (O,N)-donor chelating ligands (mono-anions of 8-oxyquinoline and of *N*,*N*-dimethylaminoethanol). They reveal that chelators with the N-C-C-O sequence can bind to the tetrels with the formation of five-membered ring motifs and that they can enhance the tetrel coordination number to, e.g., five, six or seven. Moreover, as the pair of complexes **IX** and **X** shows, formation of ionic tetrel complexes must be taken into consideration as well, and pair **X**, **XI** demonstrates that isomeric coordination modes of the bidentate ligands may be encountered with otherwise related complexes.

**Figure 2:**
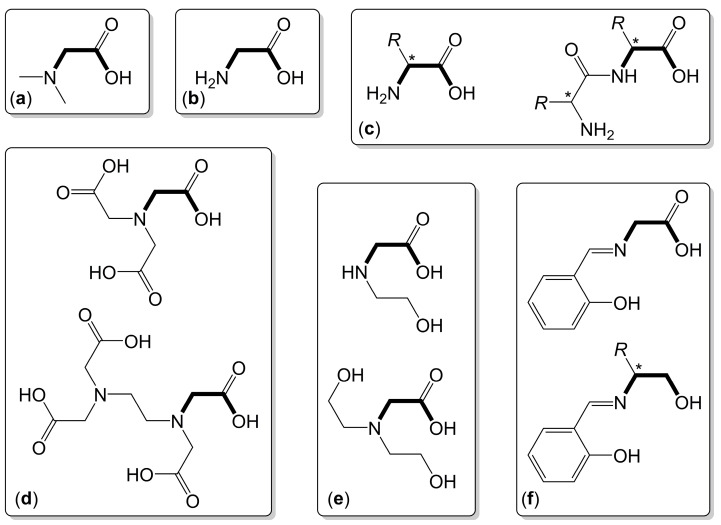
Selected (O,N)-chelators, which feature an α-amino acid motif (drawn with bold-style bonds). The fundamental features of *N*-alkylated amino acids, such as *N*,*N*-dimethylaminoacetic acid (**a**), and NH-bearing amino acids, such as glycine (**b**), may be contained in or give rise to more complex chelators such as chiral amino acids and peptides (**c**), amineoligoacetic acids (**d**) and alkanolamine acetic acids (**e**). This variety can be extended to amino acid-derived ligands, which are devoid of an amino and/or carboxylic acid group, e.g., in Schiff bases, which originate from α-amino acids (**f**). In (**c**) and (**f**), *R* represents a variety of substituents, and the asterisk (*) indicates a center of chirality. Related features may be contained in the other classes of ligands as well.

In principle, a great variety of (O,N)-chelators is available from the natural chiral pool (i.e., α-amino acids), and further chiral and non-chiral derivatives thereof (of natural or artificial origin, such as aminoacetic acid (glycine) and α-amino isobutyric acid, respectively) are available as well. Interestingly, tetrel coordination chemistry with this class of ligands has been explored only scarcely so far (vide infra), but more complex (oligodentate) derivatives of these ligands also contribute to the portfolio of tetrel complexes investigated in the past decades. In combination with our group’s ongoing investigation of (O,N)-chelate complexes of silicon [37,38,39,40,41,42,43,44], this served as our motivation to review the current state of insights into the Si-, Ge- and Sn-coordination chemistry with α-amino acid-derived chelating ligands. Figure 2a–e give a selected impression as to which types of ligands may feature an α-amino acid motif. (For the sake of simplicity, these molecules are drawn in their carboxylic acid form. We are aware that α-amino acids form zwitterionic species.) The two examples shown in Figure 2f indicate that, starting from α-amino acids, modifications at the N and/or C-terminus may give access to various other (O,N)-chelating ligands. In this regard, this review mentions compounds which feature a genuine α-amino carboxylic acid motif in the ligand backbone and compounds which lack the amine or amide motif at the N-terminus (e.g., Schiff bases) and/or the carboxylate at the C-terminus (e.g., alcoholates, phosphonates). Thus, for the sake of clarity, the former are labeled with Arabic numerals and the latter with Roman numerals. Moreover, labels of generic drawings, which resemble groups of compounds, are italicized to emphasize their variable character. The crystal structures of compounds, which are referred to in this review, can be accessed from the Cambridge Structural Database (CSD). For the readers’ convenience, the access codes (CSD reference codes) are summarized in Appendix A (Table A1, Table A2 and Table A3, for the compounds labeled with Roman numerals, with Arabic numerals and for additional compounds apart from these labeling schemes, respectively).

## 2. Si-, Ge- and Sn-Complexes of α-Amino Carboxylic Acid-Derived (O,N)-Chelating Ligands

### 2.1. Si-, Ge- and Sn-Complexes Derived from α-Amino Monocarboxylic Acids

#### 2.1.1. Si-Complexes Derived from α-Amino Monocarboxylic Acids

**Figure 3:**
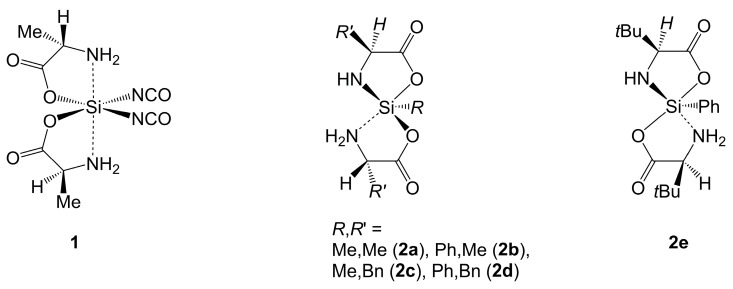
Crystallographically characterized hexa- (**1**) and pentacoordinate (**2a**–**2e**) silicon complexes, which feature a mono-anion of an α-amino acid among the (O,N)-chelating ligands. For clarity, the formally dative bond between Si and the amino group is represented by a dashed line.

Literature reports from the past decades suggest that α-amino carboxylic acids can be utilized as mono-anionic (O,N)-chelators at Si, e.g., in complexes generated by reaction of Me_3_SiCl or Ph_3_SiCl and the mono-sodium salts of amino acids such as glycine, alanine, methionine, leucine, valine or phenylalanine [45]. This series of proposed pentacoordinate silicon chelates is complemented by a series of proposed hexacoordinate silicon bis-chelates obtained in a similar manner from Me_2_SiCl_2_ and two equivalents of the amino acids’ mono-sodium salts [46]. The protic solvent used for the syntheses (i.e., methanol), however, is likely to react with the chlorosilane starting materials (i.e., formation of methoxysilanes [47]), and none of the proposed chelates has been supported by crystallographic evidence. So far, only six examples of Si-complexes with (O,N)-chelating mono-anions of α-amino acids have been characterized crystallographically (Figure 3) [48]. The authors obtained these complexes from reactions of bis(trimethylsilylated) α-amino acids and isocyanatosilanes (Si(NCO)_4_, MeSi(NCO)_3_, PhSi(NCO)_3_). The hexacoordinate Si-complex **1** derived from l-alanine features *cis*-arranged monodentate NCO groups *trans* to the chelates’ carboxylate O atoms. The two formally dative N–Si bonds are *trans* to one another. The pentacoordinate silicon complexes **2a**–**2e** feature both a mono- and a di-anionic (O,N)-chelator derived from the same amino acid. In complexes **2a**–**2d** (l-alanine and l-phenylalanine used as ligands), the NH_2_ group of the mono-anionic chelator occupies an axial position in the almost trigonal–bipyramidal Si-coordination sphere. In the l-*tert*-leucine derivative **2e**, both axial positions are occupied by the carboxylate groups. Selected geometric features of the Si-coordination spheres of these compounds are listed in Table 1. Poor solubility and decomposition in [D6]DMSO inhibited NMR spectroscopic investigations of the stereochemistry of the pentacoordinate Si-compounds **2a**–**2e** in solution [48].

**Figure 4:**
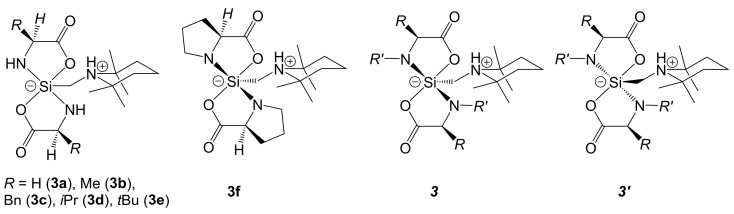
Zwitterionic spirosilicates with two di-anions of α-amino acids as (O,N)-chelators (**3a**–**3f**) [49,50]. The substituents *R*,*R*′ at N and the α-C atom in the generic drawings of the diastereomers ***3*** and ***3*′** may also represent -CH_2_CH_2_CH_2_- and thus account for the di-anion of l-proline as an (O,N)-chelator as well.

In previous studies, Tacke et al. reported on zwitterionic Si-bis-chelates, in which two di-anions of α-amino acids serve as the (O,N)-chelators (Figure 4) [49,50]. The syntheses of this class of compounds involved a dehydrogenative coupling of the amino acids with the silane H_3_Si–CH_2_–(NC_5_H_6_-2,2,6,6-Me_4_). In these compounds **3a**–**3f**, the di-anions of glycine, l-alanine, l-phenylalanine, l-valine, l-*tert*-leucine and l-proline, respectively, are employed as chelators. A common feature of these complexes is the trigonal–bipyramidal Si-coordination sphere with axial orientation of both Si–O bonds. In such a trigonal–bipyramidal arrangement, chiral amino acids (as in **3b**–**3f**) may give rise to the formation of two different diastereomers (***3*** and ***3′***). The solid-state structures reveal that the one or the other may crystallize (e.g., **3b** corresponds to type ***3***, **3c** corresponds to type ***3′***). In solution, as monitored NMR spectroscopically, pairs of these diastereomers may coexist in epimerization equilibria (as in the case of **3b**), whereas others (e.g., **3c**) do not undergo epimerization in solution at room temperature.

**Figure 5:**
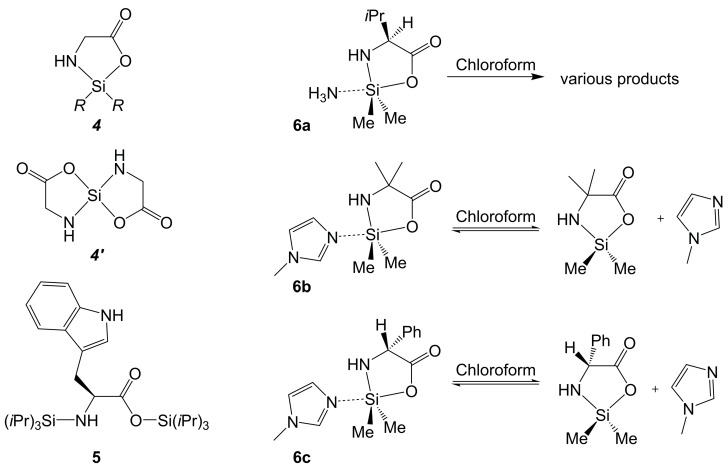
*O*,*N*-bis-silylated α-amino acids: Generic cyclic ***4*** and spirocyclic types ***4***′ (R may be various substituents, and the amino acid backbone may carry further substituents not shown in this generic sketch), bis-silylated l-tryptophan **5** [52] and structurally characterized Lewis-base adducts of silacycles of l-valine [39], α-amino isobutyric acid [37] and d-phenylglycine [37] (**6a**, **6b** and **6c**, respectively).

So far, there is no crystallographic evidence for the successful isolation of silacycles of α-amino acid di-anions with a tetracoordinate Si atom (neither cyclic ***4*** nor spirocyclic types ***4*′** in Figure 5). Nonetheless, the formation of compounds of type ***4*** as reaction intermediates has been reported in the literature [51]. In principle, silylation of the anionic C- and N-termini of the amino acid is possible, and two-fold silylated derivatives can be isolated, e.g., the bis(triisopropylsilyl) derivative of tryptophan **5** [52]. The five-membered heterocycles, however, exhibit so-called ring-strain release Lewis acidity [53], and therefore they may require a fifth donor atom in the Si-coordination sphere for further stabilization. The successful isolation and structural characterization of ammonia adduct **6a** [39] and of *N*-methylimidazole adducts **6b** and **6c** [37] are in support of the Si atom’s affinity to a fifth donor atom when incorporated in a five-membered cycle with an amino acid’s di-anion. These compounds were prepared from the respective amino acid and a silicon precursor with Si–N bonds, which either releases the required Lewis base (i.e., hexamethyldisilazane with the release of ammonia in the case of **6a**) or triggers the addition of an alternative Lewis base (i.e., starting from Me_2_Si(NH*t*Bu)_2_ and *N*-methylimidazole in the cases of **6b** and **6c**). In adducts **6a**–**6c**, the Si-coordination spheres are close to trigonal–bipyramidal, in all cases with the carboxylate O and the additional Lewis base in the axial position while the anionic N-terminus of the amino acid occupies an equatorial site. In chloroform solution, these adducts dissociate (with the formation of a variety of products in the case of **6a** [39] and in coexistence with the silacycle+NMI system in a dynamic equilibrium for **6b**, **6c** and the related l-valine derivative [37]).

Table 1 contains selected parameters of the Si-coordination spheres of the compounds mentioned in Section 2.1.1. As compound **1** is the only hexacoordinate Si-complex in this series, we cannot deduce any trends in dependence on substitution patterns. Comparison of the other 14 compounds, all of which feature pentacoordinate Si, reveals some mutual features. The distorted trigonal–bipyramidal Si-coordination spheres are founded on axial positioning of O atoms or a combination of one O atom and one non-ionic N-donor moiety. None of the complexes have any amide N atoms in the axial position. The complexes of the series **2a**–**2e** reveal the most pronounced deviation of this axis from linearity. However, there is no clear trend toward forming a square-base pyramidal coordination sphere by pronounced widening of one of the equatorial angles. Each of the compounds **2a**–**2e** features two widened equatorial angles in the range of 120.1–125.9°. The (all-equatorial) Si–N(amide) bonds of the 14 complexes were found in a rather narrow range of 1.69–1.74 Å. The (all-axial) Si–O bonds of the di-anionic chelators span a wider range (1.79–1.88 Å), which exceeds the span of the bond length range of the axial Si–N bonds to the non-ionic donor sites (1.97–2.04 Å). As to the latter, the positional change of this formally dative bond into an equatorial position (encountered with compound **2e**, 1.88 Å) has greater influence on this bond length than the variation in substitution patterns in the other 13 compounds. This shortening of the formally dative Si–N bond is accompanied by lengthening of the Si–O bond to the same chelating ligand. The resultant Si–*X* (*X* = O,N) bond lengths of the mono-anionic chelator in **2e** are similar to those encountered with the mono-anionic chelators in the hexacoordinate Si-complex **1**.

#### 2.1.2. Ge-Complexes Derived from α-Amino Monocarboxylic Acids

While in the field of Si-coordination chemistry, a series of different classes of complexes with mono- and/or di-anionic α-amino acid-derived (O,N)-chelate complexes have been reported in the past two decades, there is no crystallographic evidence for the isolation of related Ge complexes. Moreover, no monodentate binding modes of an HN–C–C(=O)O motif to Ge are yet represented in the Cambridge Structural Database (CSD). Nonetheless, some amino acid-derived germacycles were reported by Lavayssiere et al., e.g., the Et_2_Ge derivatives of the di-anions of valine and of α-amino isoburyic acid [54,55]. The authors report the use of Et_2_GeCl_2_ and a supporting base (Et_3_N) as well as the route via aminogermane Et_2_Ge(NEt_2_)_2_ for syntheses of that kind of germacycle. In a very recent report, Ignatyev et al. mentioned the formation of the compound (glycinate)Ge(OH)_3_, prepared from GeO_2_ and glycine, in which the Ge atom is chelated by a mono-anion of glycine [56].

#### 2.1.3. Sn-Complexes Derived from α-Amino Monocarboxylic Acids

**Figure 6:**
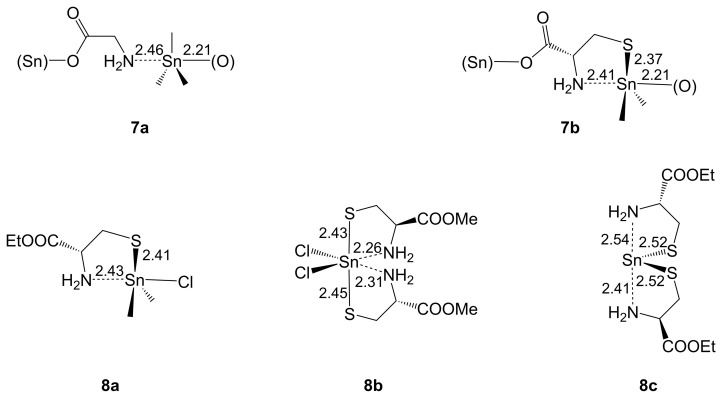
Sn(IV)-complexes of the amino acids glycine (**7a**) and l-cysteine (**7b**), as well as Sn(IV) (**8a**, **8b**) and Sn(II) (**8c**) complexes of cysteine alkyl esters. The Sn–N as well as Sn–O and Sn–S (if applicable) bond lengths (Å, rounded to two decimal places, where their s.u.s for the 2nd decimal place are (3) for **7a** and ≤(1) for the other compounds) are listed next to the respective bond. In the drawings of **7a** and **7b**, the atoms (Sn) and (O) in parentheses represent the respective atoms of adjacent molecules in the crystal structure.

As found for Ge, the CSD currently lacks entries for (O,N)-chelate complexes of Sn with mono- and/or di-anionic α-amino acid-derived ligands. However, Ho et al. reported the structure of trimethyltin glycinate **7a** [57] (Figure 6), obtained from Me_3_SnOH and glycine upon azeotropic removal of the water formed in their reaction. In this polymeric compound, the Sn atom is pentacoordinate because of the two-fold monodentate (and thus bridging) coordination mode of the amino acid’s mono-anion. In a related manner, the (O,S)-di-anion of cysteine operates as a bridging ligand in compound **7b** [58]. **7b** and related derivatives of l-cysteine (with SnEt_2_, SnBu_2_, SnPh_2_ and SnBn_2_ moiety) were prepared from the respective diorganotin dichloride, l-cysteine and NaOEt. In contrast to **7a**, compound **7b** features a five-membered chelate about the Sn atom, but this (S,N)-bidentate binding mode is special to the anion of this particular amino acid. Some related chelates of Sn(IV) (**8a** [59], **8b** [60]) and Sn(II) (**8c** [61]) were reported for mono-anions of cysteine esters. Even though they are somewhat out of scope (lacking the (O,N)-chelating motif), they allow for a comparison of the Sn-coordination of the amino acid’s NH_2_ group. Comparison of **7a** and **7b** shows that the chelate and the presence of S (instead of the third C atom) in the trigonal–bipyramidal Sn-coordination sphere strengthen the H_2_N–Sn-coordination to some extent, whereas the *trans*-disposed Sn–O bonds are similar in both compounds. Compound **8a**, albeit featuring an axial Sn–Cl bond, resembles a cutout of the structure of **7b** with similar bond lengths associated with the (N,S)-chelate at Sn. Comparison of **8a** and **8b** indicates that enhanced Lewis acidity of the Sn center (Sn-bound Me groups replaced by Cl and another (N,S)-chelate) strengthens the coordinative H_2_N–Sn bonds. Longer Sn–N and Sn–S bonds are observed with the related Sn(II) compound **8c**.

Even though there is no crystallographic analysis in support of the (O,N)-chelating properties of α-amino acid mono-anions at tin, some stannacycles of that kind were reported in the literature. Djurdjevic and Djokic reported complexation of Sn(II) by glycinate [62], and Nath and Yadaf reported the (O,N)-chelate of the mono-anion of methionine at SnMe_3_ [63]. In the context of the known polymeric structure of (glycinate)SnMe_3_ (**7a**) [57], a detailed elucidation of the coordination features of these two compounds is desirable for comparison. Moreover, related chelates were reported for other stannane and amino acid combinations by Cashion et al. [64] and Nath et al. [65].

#### 2.1.4. Concluding Remarks Regarding Si-, Ge- and Sn-Complexes Derived from α-Amino Monocarboxylic Acids

In the course of the past two decades, structural studies of Si-complexes of amino acids revealed that this portfolio of compounds is capable of binding to Si in a chelating manner, both as a mono- and as a di-anionic (O,N)-chelator. In contrast, crystallographic characterization of tin compounds of simple amino acids dates back more than four decades, and yet the structural data available to date are still very scarce. Besides the mono-anion of glycine, which was reported as a bridging ligand between SnMe_3_ groups, only cysteine and derivatives thereof were found to chelate the Sn atom (both Sn(IV) and Sn(II)) in a (S,N)-bidentate fashion. In all cases, the amino group of the ligand operates as a neutral donor moiety. Structural evidence of simple α-amino acid anions in (O,N)-chelates at Sn is yet to be delivered. This and the current lack of corresponding structural data for related Ge compounds clearly mark out some fields for further exploration. In principle, bidentate (O,N)-chelating motifs with anions of simple α-amino acids should be feasible at the heavier tetrels as well. Even though Sn is larger and more thiophilic than Si, some studies of the even heavier and more thiophilic Pb(II) (i.e., compounds Pb(leucinate)(NO_3_) [66], Pb(phenylalaninate)_2_ [67], Pb(prolinate)_2_ [68] and Pb(aspartate) [69], cf. entries **A3a**, **A3b**, **A3c** and **A3d**, respectively, in Table A3) have clearly shown that mono-anions of various α-amino acids can be bound to Pb as (O,N)-chelators, thus being capable of binding at the smallest, i.e., Si(IV) (e.g., in compounds **1** and **2a**–**2e**), and the largest, i.e., Pb(II), of the heavier tetrels. The particularly high stability of Pb(II) and the therefore possible syntheses in aqueous solution with no risk of oxidation of the tetrel and the availability of Pb(II) salts with weakly coordinating anions such as nitrate or perchlorate may have contributed to the success of the exploration of this field of coordination chemistry from this end of the tetrel series, because amino acids (as zwitterionic compounds with good solubility in water) may represent good reactants in an aqueous medium, in particular. The (O,N)-chelation by di-anionic amino acid-derived ligands was reported for Si and Ge (even though representatives of the latter still require detailed structural elucidation). In Sn-coordination chemistry, the (O,N)-di-anions of amino acids still need to be explored as ligands.

Apart from structural elucidation and gaining additional information about the tetrel coordination behavior of mono- and di-anions of simple α-amino acids, Cota et al. pointed out that complexes such as **2a** and **2c** (which comprise a SiMe motif and amino acid ligands as hydrolysable groups) may be interesting precursors for methylsilanetriol (MeSi(OH)_3_) as a food supplement [48]. Moreover, the combination of amino acid-derived chelators and amines at the same Si atom (as in **6a**) requires further exploration as starting materials for syntheses of the respective α-amino carboxylic amides [39].

### 2.2. Si-, Ge- and Sn-Complexes Derived from Dipeptides

#### 2.2.1. Si-Complexes Derived from Dipeptides

**Figure 7:**
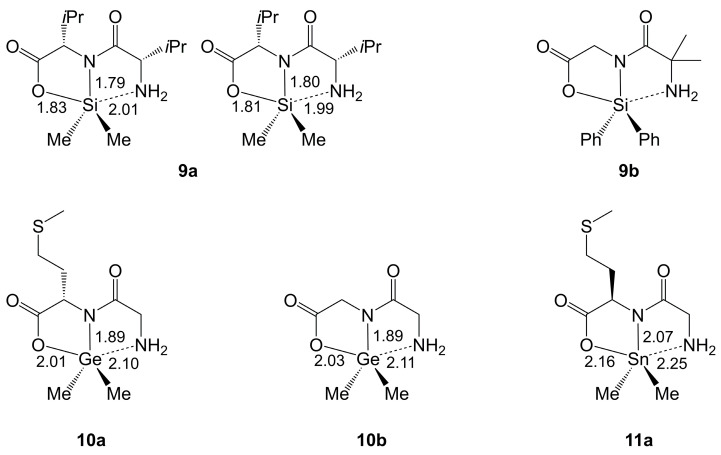
Crystallographically characterized Si-complexes of di-anions of valinylvaline (**9a**) and α-aminoisobutyrylglycine (**9b**), Ge-complexes of di-anions of glycylmethionine (**10a**) and glycylglycine (**10b**) and, as a representative example, the related SnMe_2_ complex of glycylmethionine (**11a**). Selected bond lengths (Å, rounded to two decimal places, where their s.u.s for the 2nd decimal place are <(1)) are listed next to the respective bond. For compound **9a**, the values are listed for the two crystallographically independent molecules. For compound **9b**, which contains six molecules in the asymmetric unit, the bond lengths vary in the ranges 1.80–1.82 Å for Si–O, 1.75–1.77 Å for the equatorial and 2.02–2.05 Å for the axial Si–N bond.

In 2021, the first structure of an organosilicon complex with a di-anionic homodipeptide ligand was published [39]. This compound **9a** (Figure 7) formed in a triethylamine supported reaction of Me_2_SiCl_2_ and l-valine and represents a Si-templated synthesis of a dipeptide from an unprotected amino acid. In 2022, a crystallographic analysis of a related compound (**9b**) was published by Hattori and Yamamoto. The heterodipeptide ligand of compound **9b** was deliberately synthesized in the Si-coordination sphere by using Ph_2_Si(imidazolyl)_2_, α-amino isobutyric acid and the *t*Bu-ester of glycine in a Ta(OEt)_5_-catalyzed manner [51]. The authors reported the suitability of this route for the deliberate formation of a great variety of Si-dipeptide complexes. Cota et al. already mentioned the formation of peptides as a side-reaction when utilizing silylated amino acids as starting materials (in the context of their preparation of compounds **1** and **2a**–**2e**) [48].

In compounds **9a** and **9b**, the Si atoms are situated in distorted trigonal–bipyramidal coordination spheres with the Si–O bond and the formally dative Si–NH_2_ bond in axial positions. In this regard, they are related to compounds **2a**–**2d** and **6a**–**6c**. The axial angles (165.2 and 165.4° in **9a**, 166.4–167.6° in **9b**) exhibit slightly greater deviation from linearity than in compounds **2a**–**2d**, which can be attributed to the tridentate (O,N,N)-chelate with two five-membered rings about Si. The lengths of the axial Si–N bonds correspond very well to the related axial bond lengths of compounds **2a**–**2d** and **6a**–**6c**. The di-anionic motif in compounds **9a** and **9b**, however, exhibits shorter axial Si–O bonds than in **6a**–**6c** and longer equatorial Si–N bonds (with respect to **2a**–**2d** and **6a**–**6c**).

#### 2.2.2. Ge-Complexes Derived from Dipeptides

Crystallographic studies of related germanium complexes with di-anionic dipeptide ligands were reported for compounds **10a** [70] and **10b** [71], and further compounds (such as (gly-ala)GeMe_2_ [71]) were prepared in a similar manner, i.e., from Me_2_GeBr_2_ and the free dipeptide in toluene with Et_3_N as a sacrificial base. Selected bond lengths of their Ge coordination spheres are shown in Figure 7. The coordination spheres of Ge in **10a** and **10b** are essentially related to those of Si in **9a** and **9b**. The larger covalent radius of Ge causes a more pronounced deviation of the axial angle from linearity (O-Ge-NH_2_ 161.8(1)° in **10a**, 164.4(2)° in **10b**). Interestingly, the different bonds of the dipeptide ligand respond differently to the change to the heavier tetrel. Whereas both the axial and the equatorial Si–N bonds are ca. 0.1 Å longer in the Ge compounds, the Ge–O bonds are ca. 0.2 Å longer.

Another class of Ge-complexes with dipeptide ligands (GeCl moiety with a mono-anionic and a di-anionic glycylglycine-derived ligand) has been reported by Giuffrida et al. [72]. The authors studied the molecular conformation of this compound with the aid of computational methods.

#### 2.2.3. Sn-Complexes Derived from Dipeptides

**Figure 8:**
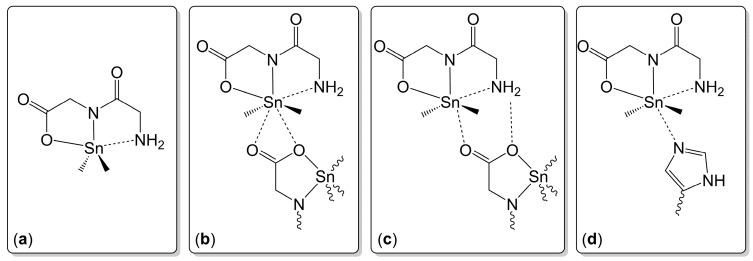
Generic representation of the Sn-coordination modes encountered in the crystal structures of dipeptide-derived diorganotin(IV) compounds (substituents at the dipeptide backbones and at the Sn-bound C atoms, if any, are omitted, and adjacent molecules are represented by the essential coordinating moiety only). (**a**) Pentacoordination of Sn (distorted trigonal–bipyramidal) with O(axial)-N(equatorial)-NH_2_(axial) positioning of the di-anionic dipeptide-derived ligand and Sn–C bonds in equatorial positions. (**b**) [5 + 2]-coordination, where two remote O⋯Sn interactions with a carboxylate moiety of an adjacent molecule enhance the Sn-coordination number in the idealized Sn(O,N,N) plane. (**c**) [5 + 1]-coordination, where one remote O⋯Sn interaction with a carbonyl O atom of the carboxylate moiety of an adjacent molecule enhances the Sn-coordination number in the idealized Sn(O,N,N) plane. The other O atom of this carboxylate group may be involved in H-bonding with the NH_2_ group (as indicated by an additional dashed line). (**d**) [5 + 1]-coordination, where one remote N⋯Sn interaction with an imidazole N atom of a histidine group of an adjacent molecule enhances the Sn-coordination number in the idealized Sn(O,N,N) plane.

In contrast to the few examples of structurally characterized dipeptide complexes of Ge, a great variety of related diorganotin complexes has been synthesized (and investigated crystallographically). The syntheses started from the free dipeptide, and the synthesis routes can be divided into three general approaches: (A) reaction of a dipeptide and a diorganotin oxide with formation of water, (B) reaction of a dipeptide and a diorganotin dialkoxide with liberation of the respective alcohol, (C) reaction of a dipeptide with sodium alkoxide (for in situ preparation of the dipeptide di-anion with liberation of the respective alcohol) and a diorganotin dichloride with formation of NaCl. As a representative example, compound **11a** [73] is listed in Figure 7. In this particular compound, the Sn atom is also coordinated in a distorted trigonal–bipyramidal manner, and with a more pronounced position of the heavier tetrel out of the tridentate (O,N,N)-chelate clamp (the axial angle is 153.0(2)°). With respect to the related Ge compounds, the formal move out of the chelate is accompanied by rather proportional lengthening of the three bonds to the chelating ligand. The variety of dipeptide-Sn-complexes, which were structurally characterized in the course of these studies, revealed a portfolio of tin coordination patterns in their solid-state structures, which depend on the absence vs. presence of additional intramolecular coordination interactions (Figure 8). They range from tin pentacoordination in a distorted trigonal–bipyramidal fashion (Figure 8a, as in **11a**), via [5 + 2]- or [5 + 1]-coordination of two O atoms or one O atom of an adjacent carboxylate group (Figure 8b,c, respectively) to [5 + 1]-coordination by a nitrogen atom of an adjacent histidine moiety (Figure 8d).

Table 2 contains selected bond lengths and angles of compounds **11a**–**11p** [73,74,75,76,77,78,79,80,81,82,83,84,85]. The features of the Sn-coordination spheres of the pentacoordinate Sn-compounds of this series are related to those of **11a**. Additional remote [5 + 1]- or [5 + 2]-coordination, however, caused pronounced widening of the C-Sn-C angle in most cases (less pronounced in **11k**). Interestingly, with respect to bonds to the tridentate dipeptide ligand, the increase in Sn-coordination number mainly causes lengthening of the formally covalent bonds (of Sn–O, in particular), whereas the formally dative bond to the NH_2_ group is less affected. We attribute this lengthening of the Sn–N bond to the fact that the increase in coordination number occurs in this equatorial plane where Sn–N is located. Pronounced lengthening of Sn–O rather Sn–NH_2_ on the O-Sn-NH_2_ axis indicates a pronounced affinity of Sn atom of these compounds to the coordination of the amino group. This is also supported by the fact that the Sn–NH_2_ bonds in compounds **11a**–**11p** are markedly shorter (by ca. 0.2 Å) than the corresponding bonds in the pentacoordinate Sn(IV) compounds **7a**, **7b** and **8a**. The remote [5 + 1]- or [5 + 2]-coordination in this class of compounds can be interpreted as an effect in the solid state. Solution-state ^119^Sn NMR data of compounds such as **11j** (*δ* ^119^Sn–198.1 ppm) and **11k** (*δ* ^119^Sn –200.7 ppm) indicate pentacoordination of the Sn atoms (in this case, in [D6]DMSO solution) [81]. In deuterated methanol, Sn pentacoordination is retained as well, as shown for **11b** (*δ* ^119^Sn–160.6 ppm) [74] as well as **11m** (*δ* ^119^Sn–175.9 ppm) and **11n** (*δ* ^119^Sn–183.4 ppm) [83].

This set of structurally characterized diorganotin compounds with (O,N,N)-chelating dipeptide ligands is complemented by further organotin compounds of amino acids and dipeptides, e.g., [86,87,88]. The particular interest in this class of compounds arose from the physiological activity of many organotin compounds, which made these combinations with bio-related molecules (i.e., dipeptides) attractive candidates for the exploration of their, for example, anti-tumor [76,83] anti-bacterial [81] and anti-inflammatory activity [80].

#### 2.2.4. Concluding Remarks Regarding Si-, Ge- and Sn-Complexes Derived from Dipeptides

**Figure 9:**
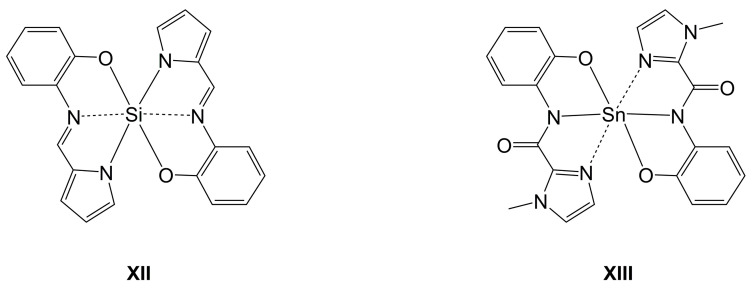
Examples of hexacoordinate Si- and Sn-complexes (**XII** and **XIII**, respectively) with two tridentate di-anionic (O,N,N)-chelating ligands and chelation through exclusive formation of five-membered rings.

Regarding the structural motifs encountered with di-anionic ligands of peptides at the heavier tetrels, only compounds with a single (O,N,N)-ligand chelating the tetrel have been reported so far. The ligands’ di-anionic nature in combination with the stability of *E* = Si(IV), Ge(IV), Sn(IV) and these tetrels’ capability of forming stable hexacoordinate complexes, however, should allow for access to dipeptide complexes of the type *E*(O,N,N)_2_. Even though structural evidence is scarce, some representatives of *E*(O,N,N)_2_ with other tridentate di-anionic chelators capable of chelating via the formation of five-membered rings only (e.g., the compounds **XII** [89] and **XIII** [90] shown in Figure 9) serve as motivation for the exploration of such complexes with dipeptide-derived ligands as well.

Moreover, to our knowledge, structural characterization of Si-, Ge- or Sn-complexes of anions of longer oligopeptides has not been reported so far. However, Hattori and Yamamoto [51] reported the utilization of silicon dipeptide complexes as starting materials for selective peptide chain elongation. This provides a perspective on a way to access and characterize silicon complexes of longer oligopeptides. Also, the utilization of silicon compounds as templates for the deliberate syntheses of peptides from unprotected [39,91] or protected amino acids [51] is a very young and promising field of amino acid coordination chemistry at heavier main group elements. In addition to organosilicon compounds, organoaluminium compounds appear to be useful tools in peptide syntheses as well [92].

From the viewpoint of coordination chemistry, the special amino acid motif of proline, which should exhibit different coordination features when incorporated into the dipeptides’ N-terminus positions because of its cyclic amine moiety, still warrants exploration.

### 2.3. Si-, Ge- and Sn-Complexes Derived from Amineoligoacetic Acids and Related Hydroxyalkylamineacetic Acids

#### 2.3.1. Si-Complexes Derived from Amineoligoacetic Acids and Related Hydroxyalkylamineacetic Acids

**Figure 10:**
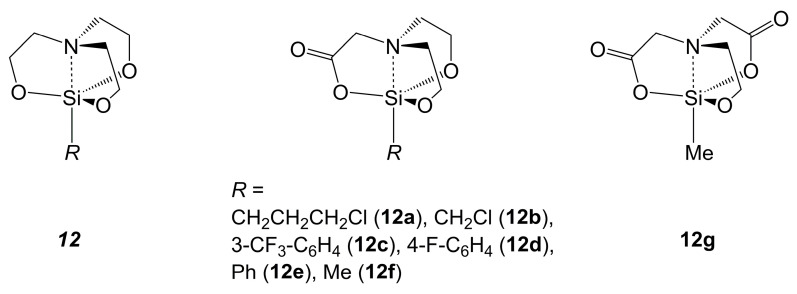
Generic structural motif of silatranes and various derivatives thereof (***12***), and listing of the crystallographically characterized silatranones (**12a** [93], **12b** [94], **12c** [95], **12d** [95], **12e** [96] and **12f** [97]) and silatranedione **12g** [98].

Silatranes (general motif ***12*** in Figure 10) represent an old and well-reviewed class of silicon complexes derived from triethanolamine. Substitution of one, two or three alcoholate groups with carboxylate groups leads to the classes of silatranones, silatranediones and silatranetriones, respectively. The molecular structures of silatranones **12a**–**12f** [93,94,95,96,97] and silatranedione **12g** [98] were analyzed by X-ray crystallography, and the molecules were found to resemble the molecular architecture of related silatranes. For example, in methylsilatrane [99] and phenylsilatrane [100], the Si–N bond lengths are 2.16 Å, the Si–O bond lengths are in the range of 1.65–1.68 Å and the sum of O-Si-O angles amounts to 356°. The ranges of Si–N bond lengths (spanning 2.09 Å in **12b** and 2.15 Å in **12a**) are very similar, and the sums of O-Si-O angles (spanning 355° in **12c** and 357° in **12b**) are very similar, too. Even though the Si–O bonds are located in similarly planarized equatorial environments around the Si atoms, their bond lengths are noticeably different for the alkoxy- vs. carboxy-O atoms. The former span a range of 1.64–1.66 Å, whereas the latter are longer (in the range of 1.71–1.72 Å in compounds **12a**–**12f** and 1.70 Å in silatranedione **12g**). The equatorially situated Si–O(carboxylate) bonds, however, are markedly shorter than the axial Si–O(carboxylate) bonds in the compounds listed in Table 1 and compounds **9a** and **9b**. Silatranetriones have also been reported [101], but their molecular structures have been analyzed by spectroscopic means only. Their ^29^Si NMR shifts (e.g., *δ* –135.8 ppm for methylsilatranetrione N(CH_2_COO)_3_SiMe in [D6]DMSO, whereas the corresponding methylsilatrane N(CH_2_CH_2_O)_3_SiMe, methylsilatranone (**12f**) and methylsilatranedione (**12g**) have ^29^Si NMR shifts of *δ* –69.7, –73.2 and –77.2 ppm, respectively, in the same solvent [101]) indicate a higher coordination number of their Si atoms, and the authors attributed this to the additional coordination of a solvent molecule. The presence of a single set of ^1^H and ^13^C NMR signals for the three CH_2_COO moieties indicates rapid site exchange of the coordinated solvent [101]. A detailed elucidation of their molecular structures is yet to be delivered to allow for discussion of the effects of solvent coordination on the bond lengths and angles of the atrane cage.

Structural analyses of quasisilatranones and quasisilatranediones (i.e., silatranone-related Si-compounds with tridentate (O,N,O)-chelators with a central NH moiety) have not been reported so far. Moreover, we are not aware of reports of silicon complexes of chelating anions derived from ethylenediamine tetraacetic acid, diethylenetriamine pentaacetic acid or related extended versions of nitrilotriacetic acid.

#### 2.3.2. Ge-Complexes Derived from Amineoligoacetic Acids and Related Hydroxyalkylamineacetic Acids

**Figure 11:**
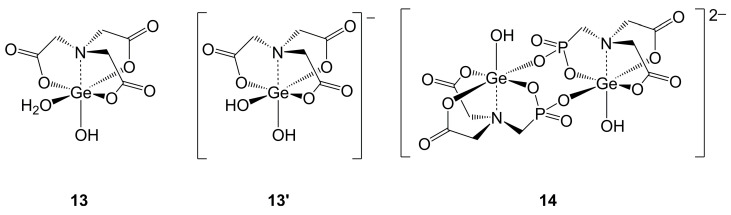
Crystallographically characterized germatranone **13** [102], its anionic relative **13′** (in the potassium salt) [103] and **14** [104]. The counter-ion in compound **14** is piperazinium.

In sharp contrast to the structural information available in the field of silatranone-type silicon complexes, the current state of crystallographic characterization of related Ge compounds lies beyond the Si congeners, with no overlap that would allow for a direct comparison of molecular structures. Even though germatranones, germatranediones and germatranetriones have been reported in the literature [101], only one crystal structure has been reported out of this group of compounds, i.e., the hydrate of hydroxygermatranetrione **13** [102] (Figure 11). In this complex, the Ge atom is hexacoordinated, and a water molecule serves as a sixth ligand moiety, located in the otherwise equatorial GeO_3_ plane of the atrane motif. In this compound, the length of the Ge–OOC bond *trans* to the Ge-bound water molecule (1.88 Å) is only marginally shorter than the mutually *trans* Ge–OOC bonds (1.89 and 1.90 Å). They are closer to the length of the dative Ge–OH_2_ bond (1.92 Å) than to the length of the formally covalent Ge–OH bond (1.76 Å). The structure of the corresponding anionic complex **13′** (K^+^ salt, two OH groups bound to Ge) has been reported as well [103]. The conformation of this anion resembles the molecular shape of **13**. The stronger coordination of the additional OH group *trans* to the carboxylate (Ge–OH bond length 1.82 Å vs. Ge–OH_2_ bond length 1.92 Å in **13**) causes particular lengthening of the *trans*-disposed Ge–OOC bond (1.97 Å), while the mutually *trans* Ge–OOC bonds (1.91 Å) remain shorter. The related compound **14** features a phosphonic acid moiety in one of its equatorial atrane positions (with respect to the N-Ge-OH axis) [104]. In this case, the anionic phosphonate serves as a ligand, which enhances the Ge coordination number to six by dimerization of this anionic complex via Ge-O-P-O-Ge bridges. The two Ge–OP bonds (1.87 and 1.88 Å) are shorter than the two Ge–OOC bonds (1.90 and 1.93 Å). The Ge–OH bond (1.77 Å) is similar to the corresponding bond in **13**. The Ge–N bonds are very similar in compounds **13**, **13′** and **14** (2.08, 2.08 and 2.09 Å, respectively).

**Figure 12:**
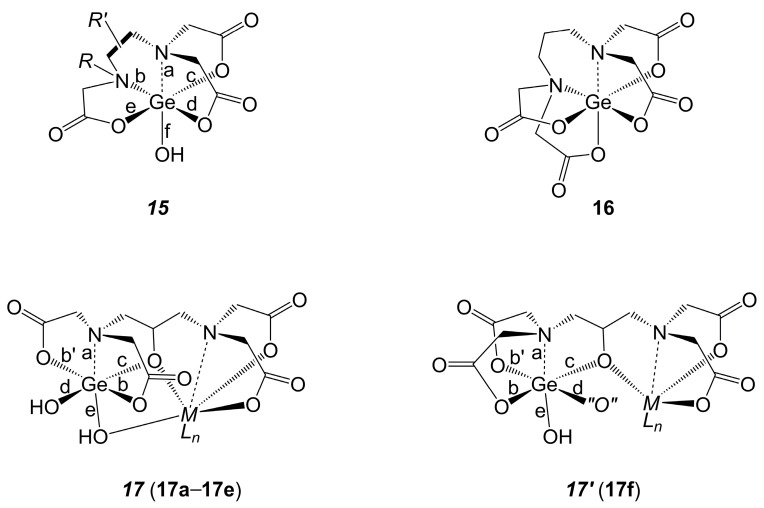
Generic drawing of germanium complexes of type ***15*** derived from EDTA and related acids with C_2_-backbone *R*′ [106,107,108,109,110] (for details of *R* and *R*′, see Table 3), complex **16** derived from a diamine tetraacetic acid with a C_3_ backbone [111] as well as examples of Ge-containing heteronuclear complexes derived from 2-propanol-1,3-diamine tetraacetic acid (***17*** [103,113,114,115,116] and ***17*′** [117,118]) (for details of *ML_n_* and “*O*”, see Table 4 and descriptions in the Discussion). Note: In each case, only one enantiomer is drawn as a representative example. In compound **16**, the Ge–N, Ge–O(*trans*-N) and Ge–O(*trans*-O) bond lengths are 2.05, 1.84 and 1.88 Å, respectively.

Moreover, detailed structural analyses are available for Ge-complexes derived from oligoamine–oligoacetic acids. This class of compounds is accessible in acidic aqueous medium from GeCl_4_ [105]. Most of them feature the ethylenediamine tetraacetic acid motif (compounds ***15*** [106,107,108,109,110], Figure 12, Table 3), and one of them has a propane-1,3-diamine backbone (compound **16** [111]). This difference, i.e., extension of the ligand backbone by one additional carbon atom, allows the diamine tetraacetate to bind to the Ge atom in a hexadentate manner, as shown in Figure 12 for compound **16**, whereas in compounds of type ***15****,* the diamine oligoacetate ligand always binds in a pentadentate manner, leaving a dangling ligand arm *R*, while an OH group occupies the sixth ligand site at Ge. As shown in Table 3, the seven representatives of compound class ***15*** (**15a**–**15f** [106,107,108,109,110], where the crystal structure of **15e** features two independent molecules in the asymmetric unit) exhibit very similar bond length patterns on their Ge coordination spheres. There is a trend that the Ge–N bond **a** of the atrane-like N atom (which features three Ge-binding arms) is similar to those in compounds **13**, **13′** and **14**, whereas the Ge–N bond **b** of the N atom with a dangling substituent is slightly longer. As bond **a** is *trans* to a more tightly bound anionic O-donor than bond **b** (i.e., bond **f** is shorter than bond **d**), we attribute the shortness of bond **a** (and the similarities of this bond length to those in compounds **13**, **13′** and **14**) to the bond-enforcing action of the tripodal ligand motif in atranes and related compounds. These interpretations are consistent with the bond lengths observed in compound **16**, which features shorter Ge–N bonds. In **16**, both N atoms are part of tripodal ligand motifs, and the Ge-coordination sphere is devoid of such a tightly bound O-donor site (i.e., OH) as in compounds of type ***15***, which would compete with the *trans*-disposed Ge–N bond. If in compounds of type ***15*** the dangling arm *R* is a chelator such as CH_2_CH_2_N(CH_2_COO^−^)_2_ (i.e., ***15*** being a derivative of diethylenetriamine pentaacetic acid), additional cations can be added to this remote ligand site with the formation of related heteronuclear complexes, such as [“*Ge*”-CH_2_CH_2_N(CH_2_COO)(CH_2_COOH)]_2_Cu, with “*Ge*” being Ge-complex motif ***15*** with the *R′* = CH_2_CH_2_ backbone [112] (cf. entry **A3e** in Table A3). A different kind of heteronuclear germatranone originates from 2-propanol-1,3-diamine tetraacetic acid (i.e., from the 2-OH functionalized derivative of the ligand used in **16**). This ligand gives rise to the formation of germatranedione cages with the alcoholate ligand arm bridging the Ge site and the adjacent heterometallic site in complexes of types ***17*** and ***17′***. In motif ***17*** (encountered with lanthanide complexes **17a**–**17e** [103,113,114,115,116]), the alcoholate donor site at Ge is *trans* to a Ge–OH bond, and the two Ge–OOC bonds are mutually *trans*. In type ***17′****,* the two Ge–OOC bonds are mutually *cis*, and another equatorial O-donor ligand (*“O”*) is bound in one of the *cis* positions next to the alcoholate. In compound **17f**, *“O”* is a water molecule. In *ML_n_* = Cu(bipy) [117] and Zn(H_2_O)_2_ complexes [118] (cf. Table A3 entries **A3f** and **A3g**, respectively) of a more complicated multinuclear architecture, a carbonyl O atom of an adjacent complex moiety occupies this position. The Ge–N, Ge–OOC and Ge–OH bond lengths in the complexes **17a**–**17f** (Table 4) are longer than corresponding bonds in germatranetrione **13**.

#### 2.3.3. Sn-Complexes Derived from Amineoligoacetic Acids and Related Hydroxyalkylamineacetic Acids

**Figure 13:**
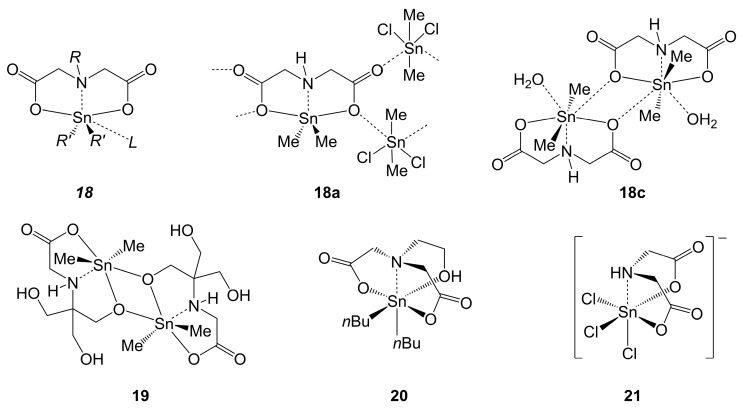
Generic drawing of tin complexes ***18*** as well as examples **18a** and **18c** (for details of *L*, *R* and *R*′ in **18a**–**18g**, see Table 5), related alcoholate bridged dimeric complex **19** [126] and complexes **20** [127] and **21** [128] with related ligands. The counter-ion in the crystal structure of the anionic complex **21** is piperazinium. Note: The Sn atoms of the bridging Me_2_SnCl_2_ moieties in **18a** are located in distorted octahedral coordination spheres.

In contrast to the Si- and Ge-chemistry shown in Section 2.3.1 and Section 2.3.2, respectively, structural characterization of tin complexes of amineoligoacetic acid derivatives has been successful with a greater variety of ligands. Particularly interesting is the set of compounds derived from diacetic acid derivatives (compounds ***18***, Figure 13). The N-bound substituent *R* may be H or hydrocarbyl (a non-coordinating residue), and for a set of diorganotin compounds derived therefrom (**18a**–**18g** [119,120,121,122,123,124,125]), two principle coordination motifs are encountered. As shown for **18a**, the di-anionic (O,N,O)-ligand was found to bind to Sn in an axial–equatorial–axial manner, and the equatorial Sn–C bonds span an angle closer to the expected 120° for equatorial positions in a trigonal bipyramid. With the aid of additional ligands *L* (such as water) and dimerization via additional remote coordination of a carboxylic O atom of an adjacent complex molecule, the Sn-coordination sphere transitions toward pentagonal–bipyramidal with axial Sn–C bonds and equatorial positioning of N and O atoms (as shown in Figure 13 for **18c**). As shown in Table 5, this adoption of pentagonal–bipyramidal coordination is similar for compounds **18c**–**18g**, all resembling a [6 + 1]-coordination sphere ([4 + 1] in the equatorial plane) with the carboxylate O of an adjacent molecule being the remote lone-pair donor. The C-Sn-C angle widening (toward axial positions) is more pronounced than in the dipeptide complexes **11e**, **11g** and **11i**, which also feature additional remote coordination in the solid state (cf. Table 2). Moreover, the related complex **19** (Figure 13, [126]) adopts an intermediate role in this regard. Its Sn atom is hexacoordinated, and its C-Sn-C angle is 141.7°. The different electronic situation in **19**, however, causes further differences, and therefore rather withdraws this complex from comparison within a series. That is, the different bridging atom (alcoholate instead of carboxylate) forms short bonds to both Sn atoms (2.07 and 2.34 Å of the intra- and intermolecular bond length, respectively), whereas the Sn–O(carboxylate) bond is rather long (2.49 Å). Other changes in the ligand (such as a coordinating substituent *R*, a 2-hydroxyethyl group) may give rise to different coordination patterns, i.e., the stannatranedione motif with hexacoordinate Sn in **20** [127]. In spite of the additional ligand arm, the Sn–N bond length in **20** (2.32 Å) is similar to those in compounds **18a**–**18g**. Whereas in compounds ***18*** and in **20** the (O,N,O)-ligand binds to Sn in a *mer*-like fashion (positioning of both O,N,O and Sn close to an idealized plane), the anionic complex **21** [128] represents a structurally characterized example of this ligand binding to octahedrally coordinated Sn in a *fac* configuration. In addition to the different configuration of the ligand and the set of electron-withdrawing substituents at Sn, the Sn–N bond in **21** (2.22 Å) is markedly shorter than those in compounds ***18*** and **20**. Extension of the ligand backbone, toward ethylendiamine diacetate (OOCCH_2_NHCH_2_CH_2_NHCH_2_COO)^2–^, also allows for the formation of octahedral Sn-complexes, as shown for the SnMe_2_ derivative, which has a *trans*-N-Sn-C and *trans*-O-Sn-O arrangement [122] (cf. Table A3 entry **A3h**).

**Figure 14:**
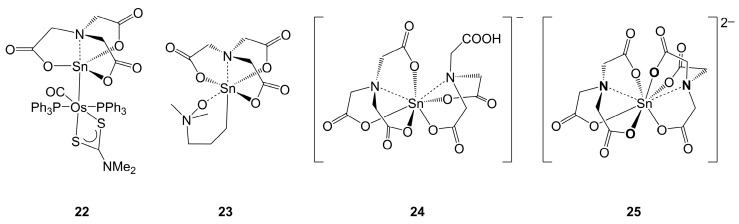
Molecular drawings of crystallographically characterized compounds with the stannatranetrione motif with a penta- (**22**) [129] hexa- (**23**) [130], hepta- (**24**) and octacoordinated Sn atom (**25**). The anionic complex **24** was characterized in its Cs salt [131], and the anion **25** was reported for its, for example, K [132], Rb [132], methylammonium [133] and guanidinium salt [133].

Using nitrilotriacetate, Sn-complexes with a penta- (**22**) [129], hexa- (**23**) [130], hepta- (**24**) [131] and octacoordinated Sn atom (**25**) [132,133] were prepared, the molecular structures of which were characterized (Figure 14). Compound **22** was prepared from the corresponding triiodostannyl complex and nitrilotriacetic acid in dichloromethane in a base-supported substitution reaction with retention of the Sn–Os bond. Compound **23** formed in a Sn–C(Ph) bond cleavage reaction of Me_2_N(CH_2_)_3_SnPh_3_ and nitrilotriacetic acid in DMF. Hence, in addition to the liberation of benzene as the expected leaving group, the NMe_2_ moiety was converted into the corresponding amine *N*-oxide, which furnishes the Sn-coordination sphere of **23**. Salts of the anions **24** and **25** were accessible in aqueous solution.

The Sn-coordination sphere in **22** is trigonal–bipyramidal, with the Sn–Os bond in the axial position and a *trans*-disposed N–Sn bond. In **23**, the tripodal ligand adopts a configuration as shown in Figure 14, which is related to the structures of compounds **20** and **21**, combining their Sn-OOC positions in one molecule. This arrangement in an octahedral Sn-coordination sphere may play an important role in the Sn–N bond length of this molecule, because this bond length is in the lower range for this set of three octahedral Sn-complexes (2.23 Å in **23**, 2.22 Å in **21**, 2.32 Å in **20**), whereas the penta-, hepta- and octacoordinated Sn-compounds in Figure 14 feature markedly longer Sn–N bonds (2.36 Å in **22**, 2.32 and 2.35 Å for the tetra- and the tridentate ligand site, respectively, in **24**, 2.37 Å in the K-salt of **25**, 2.38 and 2.40 Å in the methylammonium salt of **25**).

The molecular conformation of anion **24** can be related to the atrane-type cage (as in **22**) for the tetradentate ligand, and the three bonds to the donor atoms of the tridentate ligand are staggered about the O-Sn-O angles of the former. In contrast, in di-anionic octacoordinated Sn-complex **25**, the two tetradentate ligand moieties adopt conformations which are comparable to those in **23**, and a Sn–O bond of one ligand opposes the vacant face of the other ligand (and vice versa), as indicated by the bold-style **O** and **N** atoms in Figure 14 (vacant face at bold **N** of one ligand exposed to the bold **O** of the other ligand and vice versa).

**Figure 15:**
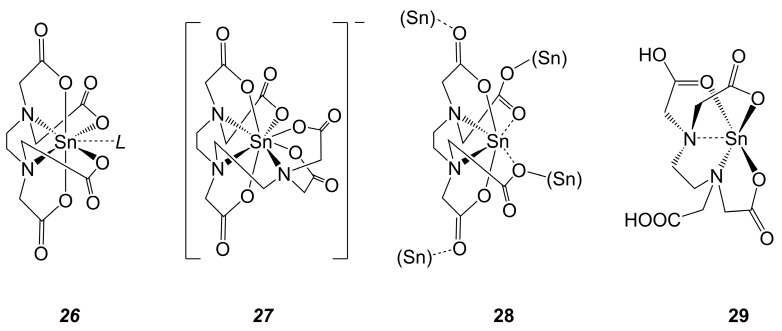
Molecular drawings of crystallographically characterized tin compounds of the anions of ethylenediaminetetraacetic acid and diethylenetriaminepentaacetic acid. In compounds of type ***26***, the ligand *L* may be formally charge-neutral (like OHSnCl_3_(H_2_O) [134] or H_2_O [135]) or anionic (like hydroxide [136,137,138], fluoride [137] or chloride [139]), resulting in non-charged or anionic Sn-complexes, respectively. Compounds of type ***27*** were reported as the hydrated protio form [140,141] and as the ammonium salt [142]. In the drawing of **28**, the atoms (Sn) in parentheses represent the additional Sn(II) sites in the crystal structure, which feature SnO_4_-coordination spheres.

This trend toward coordination numbers 7 or even 8 of the Sn atom, which contrasts the Sn-coordination chemistry from related Ge- and Si-chemistry, is also evident in Sn-complexes of oligoamine oligoacetic acid-derived ligands. Various derivatives of Sn(EDTA) (EDTA being the tetra-anion ethylenediamine tetraacetate) of the general molecular building pattern ***26*** [134,135,136,137,138,139] (Figure 15) were characterized crystallographically, and their mutual feature is a heptacoordinate Sn in a coordination sphere, which can be described as a distorted pentagonal bipyramid. As shown in Figure 15, the axial positions are occupied by carboxylate O atoms (forming the shortest Sn–O bonds in this coordination sphere), and the sets of atoms in the idealized pentagonal plane (two N atoms, two further carboxylate O atoms, one additional lone-pair donor ligand *L*) are distorted against one another in terms of a twist of the N_2_Sn plane against the SnO_2_*L* plane. This building pattern (although more distorted off the bipyramidal shape of compounds of type ***26***) is in general retained in Sn(IV) complexes derived from diethylenetriaminepentaacetic acid (motif ***27***, both the protio form [140,141] and the ammonium salt [142] were characterized crystallographically). Their building pattern is related to that of compounds ***26*** by replacing an equatorial O atom with the third amine N, accompanied by the replacement of ligand L with O atoms of the additional two carboxylate moieties. Thus, in compounds of type ***27***, the Sn atom is octacoordinated and completely encapsulated in the octadentate ligand. Also related to the molecular structures of ***26*** and ***27*** is tin(II) compound (EDTA)Sn_2_ (**28**) [143]. The solid-state structure of **28** features two different Sn(II) coordination spheres; one Sn atom is located in a SnO_4_-coordination sphere, surrounded by carboxylate O atoms (it serves as the bridging Sn site, indicated by “(Sn)” in Figure 15), whereas the other Sn atom is located in the EDTA chelate in a SnN_2_O_4_-coordination sphere (in a [4 + 2]-coordination with two markedly longer Sn-O distances *trans* to the Sn⋯N bonds), which relates to those of ***26*** by replacing the seventh ligand *L* with the Sn-located lone pair, and it relates to ***27*** in terms of the pronounced distortion off the axial geometry. In sharp contrast to **28**, tin(II) compound (EDTA)H_2_Sn (**29**) [144] adopts a tetrel coordination sphere related to those of Ge compounds of type ***15*** (the site of the Ge-bound OH group in **15** is replaced with the Sn-located lone pair in **29**).

#### 2.3.4. Concluding Remarks Regarding Si-, Ge- and Sn-Complexes Derived from Amineoligoacetic Acids and Related Hydroxyalkylamineacetic Acids

The exploration of Ge- and Sn-coordination chemistry benefits from the availability of compounds such as EDTA complexes from an aqueous medium. This allows for access to a variety of complexes which feature, for example, an atranetrione or -dione ligand backbone about Ge or Sn. Because of the size difference between Ge and Sn, different coordination modes of EDTA and related anions are encountered (vide supra). Interestingly, EDTA complexes of *hs*-Fe^3+^, which has an ionic radius between those of the formal ionic radii of Ge^4+^ and Sn^4+^ [145], include the motifs encountered with Ge (in [Fe(HEDTA)(H_2_O)] [146]) and with Sn (in K[Fe(EDTA)(H_2_O)] [147]) (cf. Table A3, entries **A3i** and **A3j**, respectively). The class of complexes presented in Section 2.3.3. comprises some structurally characterized examples of Sn(II) compounds. In this regard, it serves as a motivator for further exploration of related Sn(II) complexes with ligands mentioned in Section 2.1 and Section 2.2. Moreover, the wealth of coordination chemistry encountered with the Ge- and Sn-complexes in Section 2.3. raises questions as to the facets of Si-coordination chemistry of anions of amineoligoacetic acids beyond silatranediones, with the Si-hexacoordinate silatranetriones as an entrance into this world of coordination chemistry of interesting compounds yet to be characterized in detail.

## 3. Perspectives Outside the Box

### 3.1. α-Amino Acid-Derived Schiff Bases as Ligands

**Figure 16:**
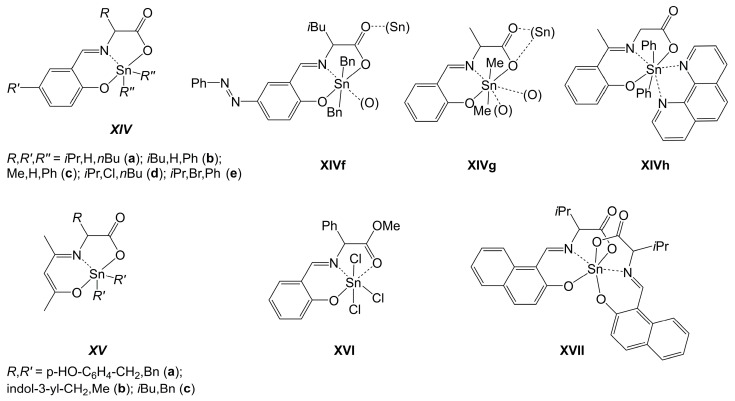
Molecular sketches of selected crystallographically characterized Sn-complexes with Schiff-base-type tridentate (O,N,O)-chelating ligands derived from α-amino acids. In compounds **XIVf** and **XIVg,** the (Sn) and (O) atoms in parentheses indicate additional coordination with the respective atomic sites of adjacent molecules in the crystal structure.

In principle, the primary amine motif of an α-amino acid motivates researchers to create related ligands by Schiff base condensation of this group with suitable carbonyl compounds (such as o-hydroxyarylcarbaldehydes, -ketones or acetylacetone). In 1992, Smith et al. reported the first crystal structure of an (O,N,O)-chelated tin compound with a Schiff base derived from an α-C substituted α-amino acid, a compound of type ***XIV*** (Figure 16, compound **XIVa** [148]). It features a di-anionic (O,N,O)-tridentate Schiff base ligand derived from salicylaldehyde and valine, and further compounds of that kind were reported in the course of this investigation. The Schiff base ligands were accessible by condensation reaction of the aldehyde and the free amino acid, and the tin complexes were prepared from the Schiff base ligands and diorganotin oxides [148]. Further compounds of that kind were reported thereafter, e.g., compounds **XIVb** [149] and **XIVc** [150] with ligands derived from leucine and alanine, respectively, and it was reported that racemization of the chiral Schiff base compounds may occur [150]. Moreover, modifications at the aryl part of the ligand backbone enhanced the portfolio of these compounds (e.g., **XIVd** and **XIVe** [151]); they were pursued as this kind of tin compound was of interest for the exploration of their cytotoxic activity. In the course of the past decade, acetylacetone-derived Schiff bases (e.g., **XVa** [152], **XVb** [153], **XVc** [154]) have enhanced the portfolio of this class of tin compounds even further (also with the aim of exploring their biological activity, e.g., for use against moscito larvae [154]). As found with other diorganotin(IV) compounds with di-anionic tridentate (O,N,O)-ligands (cf. Section 2.2.3 and Section 2.3.3), Schiff base complexes such as compounds of type ***XIV*** may establish additional intermolecular interactions, which enhance the Sn-coordination number to 6 (e.g., compound **XIVf** [155]), [6 + 1] (compound **XIVg** [156]) or 7 (in **XIVh** [157]). Within the portfolio of amino acid-derived Schiff base complexes of tin, compounds **XVI** [158] and **XVII** [159] are particularly noteworthy as they enhance our understanding of the coordination properties of this class of tridentate ligands. The former features a mono-anionic (O,N,O)-ligand, which also binds to tin in a tridentate manner. The latter represents (to date) the only crystallographically characterized tin complex which features two of these amino acid-derived Schiff base ligands. In both cases, the Sn atom is hexacoordinate, and the (O,N,O)-ligands occupy meridional positions in the octahedral Sn-coordination spheres.

**Figure 17:**
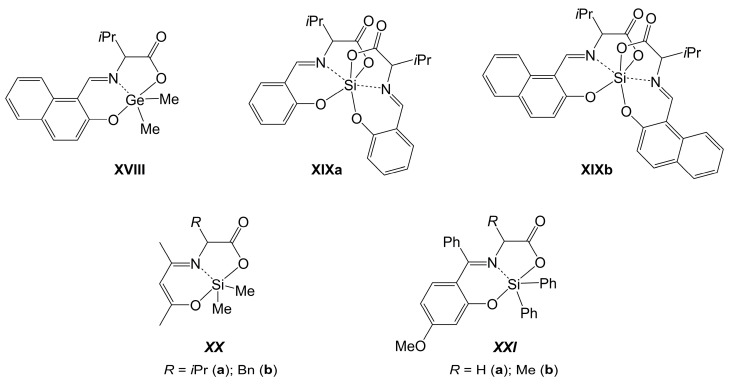
Molecular sketches of selected crystallographically characterized Ge- and Si-complexes with Schiff-base-type tridentate (O,N,O)-chelating ligands derived from α-amino acids.

In the past two decades, these studies were complemented by the investigation of some related Ge- and Si-compounds. In 2002, Nath and Goyal reported the syntheses of some organosilicon compounds with amino acid-derived Schiff base ligands and the investigation of their antimicrobial activity (even though the authors also mentioned that these compounds are sensitive to hydrolysis) [160]. Detailed insights into the molecular structures of such complexes were delivered for aldehyde-derived compounds such as **XVIII** [159], **XIXa** [161] and **XIXb** [159], acetylacetone-derived compounds such as **XXa** [162] and **XXb** [163] and even for compounds of sterically more demanding imine backbones (**XXIa** [164] and **XXIb** [165]) (see Figure 17). It was also pointed out that racemization can present an issue in case of the preparation of silicon compounds of amino acid-derived Schiff base ligands [161].

### 3.2. α-Amino Acid-Derived Alcohols as Ligands

**Figure 18:**
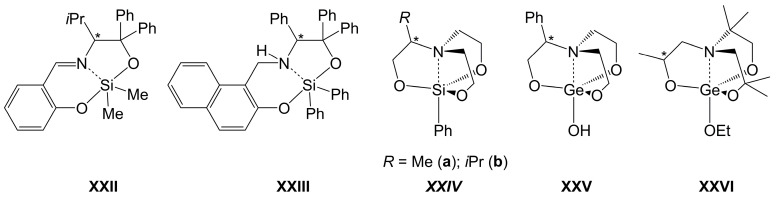
Pentacoordinate Si- and Ge-compounds with chiral chelating ligands that originate from chiral α-amino acids (**XXII** [166], **XXIII** [167], ***XXIV*** [168]) and from epoxides (**XXV** [169] and **XXVI** [170]). The asterisk (*) denotes the asymmetric C atom.

In addition to the preparation of complexes with mono- or di-anionic α-amino acid ligand motifs, amino acids (especially the chiral representatives thereof) offer great potential for transferring their chiral information to other classes of ligands. The chiral amino alcohol H_2_N-C*H*i*Pr-CPh_2_-OH, which can be obtained from valine, can be utilized for the preparation of chiral Schiff base ligands and Si-complexes thereof as well, compound **XXII** (Figure 18) being a representative thereof [166]. Compound **XXIII** represents a Si-complex with a related aminoalcohol motif in a tridentate (O,N,O)-ligand that features an amine moiety as the formal dative bond donor [167]. To name a second class of oligodentate ligands, chiral triethanolamine derivatives (and complexes thereof) may also be obtained from chiral amino acids, e.g., compounds of type ***XXIV***, which are based on alanine and valine as the chiral starting materials [168]. While other routes may also be employed (e.g., the -CHPhCH_2_O- and -CH_2_CHMeO- moieties in the ligands of compounds **XXV** [169] and **XXVI** [170], respectively, originate from ring-opening of styreneoxide and propyleneoxide, respectively), the amino acids from the natural chiral pool offer a variety of building blocks, which can be converted into different kinds of other chiral ligands.

### 3.3. Ligands Derived from Non-Carboxylic α-Amino Acids

**Figure 19:**
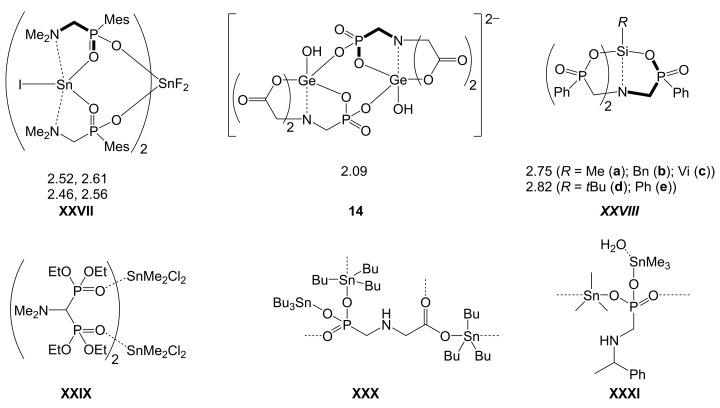
Molecular sketches of Sn-, Ge- and Si-compounds with ligands of the α-aminophosphonic acid or α-aminophosphinic acid type. For compounds **XXVII**, **14** and the set of compounds ***XXVIII****,* the *E*⋯N distances (*E* = Sn, Ge, Si) in Å are listed. For compounds **XXX** and **XXXI**, dashed lines indicate intermolecular Sn⋯O coordination in the crystal structure.

Even though silicon analogs of the type *R*_2_N-CH_2_-Si(=O)OH are not known, the α-amino carboxylic acid motif is related to other heavier main group element (*E*)-based acids of the type *R*_2_N-CH_2_-*E*(=O)*R′*OH (*R*, *R′* being various kinds of residues, e.g., alkyl, aryl, H and, for *R*′, extending to OH, alkyoxy and others) such as aminoalkylphosphonic or -sulfonic acid derivatives. For some simple representatives thereof, coordination chemistry has been explored to some extent, e.g., syntheses and characterization of the copper complexes of H_2_N-CH_2_-P*R*(=O)O^–^ (*R* = Me, Ph) [171] and H_2_N-CH_2_-S(=O)_2_O^–^ [172] (cf. Table A3, entries **A3k**, **A3l** and **A3m**, respectively), in which these mono-anions operate as (O,N)-chelators. A search for crystallographic evidence of these special kinds of α-amino acid motifs acting as chelators in Si/Ge/Sn-coordination chemistry merely yielded some hits for phosphonic and phosphinic acid derivatives (**XXVII** [173], **14** [104], **XXVIII** [174], Figure 19). Interestingly, only the Ge representative, which has N clamped to Ge by additional carboxylate buttresses, exhibits a rather short bond between the amine N atom and the heavier tetrel. The coordinative N–*E* bonds for the *E* = Sn, Si representatives are markedly longer. For the series of Si-compounds **XXVIIIa-e** shown in Figure 19, this was rather unexpected; the anion of the trifunctional aminomethylphosphinic acid (nitrilotris(phenylphosphinic acid) [175]) allowed for rather long N–Si distances in spite of the additional buttresses. Of note, the Sn representative **XXVII** features N–Sn-coordination at the Sn(II) site of this Sn(II)_2_Sn(IV) trinuclear complex. Sn-complexes of [4-HOOC-C_6_H_4_-CH_2_N(CH_2_PO_3_)_2_]^4–^ (Sn–N 2.92 Å) [176], [–CH(CH_2_CH_2_)_2_NCH_2_PO_3_^2–^]_2_ (Sn–N 2.94, 2.96, 3.23 Å; 2.81, 3.13, 3.19 Å) [177] and CyN(CH_2_PO_3_^2–^)_2_ (Sn–N 2.96 Å) [178] (cf. Table A3, entries **A3n**, **A3o** and **A3p**, respectively) contribute to the portfolio for the heavier tetrel. All of them feature amine-Sn-coordination at Sn(II) with rather long Sn–N distances (listed in parentheses). As for Sn(IV) representatives, structures of other Sn-compounds of α-amino phosphonic and phosphinic acid derivatives were reported in which the respective molecule or anion binds to Sn via O only, leaving behind a vacant N lone-pair donor site. Figure 19 shows some selected examples (**XXIX** [179], **XXX** [180], **XXXI** [181]), and the SnCl_2_Me_2_-complex of [(EtO)_2_(O=)PCH(Ph)-NH-CH_2_]_2_ [182] (cf. Table A3 entry **A3q**) represents a further example. Of note, coordination of amine N to phosphonate-functionalized Sn(IV) at a rather short distance (2.45 Å) is in principle feasible, as shown by Dakternieks et al. for compound MeN(CH_2_CH_2_CH_2_)_2_SnMe(O(OH)O=P*t*Bu) [183] (cf. Table A3 entry **A3r**).

**Figure 20:**
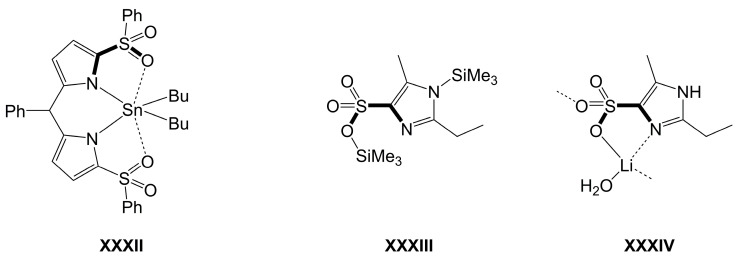
Molecular sketches of compounds which feature SO moieties inside five-membered (O,N)-Sn-chelates (**XXXII**) and which may be considered precursors for SO-functionalized (O,N)-chelating ligands (**XXXIII** and **XXXIV**).

As for sulfur analogs, compound **XXXII** shown in Figure 20 [184] represents the only crystallographically characterized compound with a five-membered (N-C-S-O)Sn-chelate motif, and representatives of Ge and Si are yet to be explored. In principle, compounds such as the SiMe_3_-derivative **XXXIII** shown in Figure 20 [185] may turn out to be suitable starting materials for syntheses of (N-C-S-O)*E* (*E* = Si, Ge, Sn)-chelates. The compound itself would be a neutral (N-C-S-O)-chelator, but the SiMe_3_ groups as leaving groups offer its transformation into an anionic chelator. In its Li salt (**XXXIV**), the anion forms a five-membered (N-C-S-O)Li-chelate [185].

## 4. Conclusions

This overview of heavier tetrel derivatives of α-amino acids, where anions of the latter operate as (O,N)-chelating ligands, demonstrates the variety of ligands with the α-amino acid motif in Si-, Ge- and Sn-coordination chemistry. Some classes of complexes (e.g., complexes of di-anions of dipeptides) were reported with structural characterization for the series of the three tetrels and thus allow for comparison of the coordination behaviors of the different tetrels. For some classes (e.g., complexes derived from amine diacetic acid), series of crystallographically characterized representatives were reported for one tetrel only (*E* = Sn), which at least allow for comparison of structural features within the set of complexes. In the context of the availability of a great variety of amino acids from the natural chiral pool, however, the portfolio of structurally characterized Si-, Ge- and Sn-complexes of these ligands is rather limited. Moreover, rather common amine oligoacetic acids (such as nitrilotriacetic acid, ethylenediamine tetraacetic acid) are also highly underrepresented in some fields of tetrel coordination chemistry as far as unequivocal characterization of the molecular structures of the resultant complexes is concerned. Some emerging fields of tetrel coordination chemistry with α-amino acid-derived ligands indicate that the overall topic of tetrel coordination chemistry with these kinds of (O,N)-chelators is a live and growing field. Just to name sections of the timeline and give some examples: The first crystallographic characterization of a tin complex of a chiral amino acid-derived Schiff base ligand dates back to 1992 [148], and examples with Si and Ge followed in 2012 [161] and 2018 [159], respectively. Whereas complexes of di-anions of dipeptides were structurally characterized as early as the 1970s for Sn [85] and 1980s for Ge [70,71], the first crystallographic report of a dipeptide-derived Si-complex was published in 2021 [39]. However, gaps in some series of classes of complexes predominate. Detailed structural characterization is yet to be delivered for, e.g., silatranetriones, which are interesting because of their tendency toward Si-hexacoordination (as indicated by ^29^Si NMR spectroscopic data) [101]; for EDTA complexes of silicon, which would complement the examples of heptacoordinate Sn- and hexacoordinate Ge-complexes of this well-known chelator; for Ge-complexes of mono- and di-anions of simple α-amino acids, where there are examples of chelating mono- and di-anions of that kind at Si [37,39,48,49,50]; and structural evidence for the bridging coordination of the glycinate mono-anion in polymeric (gly)SnMe_3_ [57], which raise questions as to the preferred chelating or bridging coordination of these ligands in Ge compounds. In addition to the apparent gaps in series of compounds, this overview sheds light on further fields of coordination chemistry yet to be explored. Tetrel coordination chemistry of other α-amino acids (such as α-amino phosphonic or phosphinic acids) is an almost blank space on the map of structurally characterized compounds. Moreover, in spite of the enhanced stability of di-valent tin (with respect to Ge(II) or Si(II)), the field of Sn(II)-compounds of α-amino acids is rather underrepresented. In this regard, chelates of the type (O,N)_2_Sn(II) with amino acid-derived mono-anionic (O,N)-chelating ligands may represent interesting ligands themselves in transition metal coordination chemistry because of the lone-pair donor capability of Sn(II). For other chelates, some transition metal complexes have been characterized, e.g., (8-oxyquinolinate)_2_GeCr(CO)_5_ [186] and (8-oxyquinolinate)_2_SnCr(CO)_5_ [187] (cf. Table A3 entries **A3s** and **A3t**, respectively). For α-amino acid-derived complexes of Sn (or even Ge or Si), however, this kind of transition metal coordination chemistry with group 14 ylene ligands is an unexplored field. So far, stannatranetrione compound **22** [129], which features an Os–Sn bond, is the only crystallographically characterized *TM*–*E* compound (*TM* = any transition metal, *E* = Si,Ge,Sn), which features an *E*-bound α-amino acid-derived ligand backbone.

## Figures and Tables

**Table 1:** Selected bond lengths (Å) and angles (deg.) of the Si-coordination spheres of compounds **1**, **2a**–**2e**, **3a**–**3f** and **6a**–**6c**. The values for each column are rounded to the same number of decimal places, and the s.u. on the last decimal place listed is ≤(1).

	Axis	Axial Angle	Si–N ^1,2^	Si⋯N ^1,2^	Si–O ^1,2^	Si–O ^1,2^
**1**	H_2_N-Si-NH_2_	172.1	n/a	1.89 ^3^	n/a	1.80 ^3^
	O-Si-(NCO)	176.8 ^3^				
**2a**	O-Si-NH_2_	167.6	1.69 ^e^	1.99 ^a^	1.79 ^a^	1.71 ^e^
**2b**	O-Si-NH_2_	168.3	1.69 ^e^	1.97 ^a^	1.79 ^a^	1.71 ^e^
**2c** ^4^	O-Si-NH_2_	169.0, 170.6	1.71, 1.71 ^e^	1.97, 1.98 ^a^	1.80, 1.80 ^a^	1.72, 1.72 ^e^
**2d**	O-Si-NH_2_	169.4	1.70 ^e^	1.97 ^a^	1.80 ^a^	1.70 ^e^
**2e**	O-Si-O	164.9	1.70 ^e^	1.88 ^e^	1.79 ^a^	1.82 ^a^
**3a**	O-Si-O	176.3	1.71, 1.72 ^e^	n/a	1.84, 1.82 ^a^	n/a
**3b**	O-Si-O	178.5	1.71, 1.72 ^e^	n/a	1.83, 1.81 ^a^	n/a
**3c**	O-Si-O	175.8	1.73, 1.73 ^e^	n/a	1.84, 1.82 ^a^	n/a
**3d** ^5^	O-Si-O	173.1–174.5	1.71–1.72 ^e^	n/a	1.81–1.83 ^a^	n/a
**3e**	O-Si-O	176.8	1.71, 1.71 ^e^	n/a	1.81, 1.83 ^a^	n/a
**3f** ^5^	O-Si-O	177.6–179.3	1.73–1.74 ^e^	n/a	1.81–1.85 ^a^	n/a
**6a**	O-Si-NH_3_	171.1	1.72 ^e^	2.01	1.88 ^a^	n/a
**6b**	O-Si-NMI	172.3	1.71 ^e^	2.04	1.85 ^a^	n/a
**6c**	O-Si-NMI	171.3	1.72 ^e^	2.01	1.87 ^a^	n/a

^1^ The bonds refer to those of the amino acids’ anions in the order Si–N to the di-anionic ligand, Si⋯N to the mono-anionic ligand, Si–O to the di-anionic ligand, Si–O to the mono-anionic ligand. ^2^ The superscript indices (^a^) and (^e^) indicate axial and equatorial positioning, respectively, of the respective donor atom (N or O) in the Si-coordination sphere ^3^ The two features of the respective entry are identical because of crystallographic symmetry. ^4^ The two lines contain entries for the two crystallographically independent molecules. ^5^ Ranges are given for the corresponding data of the multiple independent molecules of the crystal structures.

**Table 2:** Synthesis routes and selected bond lengths (Å) and angles (deg.) of the Sn-coordination spheres of crystallographically characterized tin compounds (**11a**–**11p**) of the type (*a*-*b*)Sn*R*_2_, which feature a tridentate di-anionic dipeptide-derived (O,N,N)-chelating ligand, with *a* and *b* being the C- and N-termini of the dipeptide-di-anion and *R* being the Sn-bound hydrocarbyl groups. The values for each column are rounded to the same number of decimal places, and the s.u. on the last decimal place listed is ≤(1).

	Ref.	Route	(*a*-*b*)*R* ^2^	O-Sn-N	C-Sn-C	Si–O	Si–N	Si⋯N	Motif
**11a**	[73]	B	(met-gly)Me	153.0	123.8	2.16	2.07	2.25	a
**11b**	[74]	C	(phe-tyr)Me	149.5	136.5	2.20	2.10	2.25	c ^3^
**11c** ^1^	[75]	A	(his-met)Me	147.0	124.7	2.14	2.12	2.30	a
				152.3	126.1	2.18	2.07	2.24	a
**11d**	[76]	A	(met-met)Me	148.8	132.0	2.19	2.11	2.26	c ^3^
**11e**	[76]	A	(his-ala)Me	146.4	143.9	2.22	2.14	2.28	b ^3^
**11f**	[77]	A	(ala-trp)Me	151.5	123.8	2.18	2.06	2.27	a
**11g** ^1^	[78]	A	(his-gly)Et	151.9	128.8	2.18	2.10	2.27	a
				150.0	148.5	2.24	2.13	2.27	d ^3^
**11h**	[79]	C	(tyr-gly)Et	152.2	131.4	2.19	2.08	2.29	a
**11i**	[80]	C	(gly-trp)*n*Bu	147.9	151.9	2.25	2.12	2.29	b ^3^
**11j**	[81]	A	(leu-aib)*n*Bu	148.7	127.8	2.20	2.11	2.28	a
**11k**	[81]	A	(ala-aib)*n*Bu	147.6	129.8	2.24	2.12	2.24	c ^3^
**11l**	[82]	A,B,C	(val-gly)*n*Bu	151.3	125.3	2.14	2.10	2.27	a
**11m**	[83]	A	(gly-gly)Cy	151.4	123.6	2.17	2.10	2.28	a
**11n**	[83]	A	(ala-gly)Cy	150.8	123.0	2.16	2.09	2.30	a
**11o**	[84]	C	(gly-gly)*t*Bu	149.6	121.7	2.20	2.09	2.29	a
**11p**	[85]	A	(gly-gly)Ph	153.2	117.5	2.16	2.08	2.27	a

^1^ Data listed for two independent molecules in the crystallographic asymmetric unit. ^2^ The abbreviations correspond to ala = alanine, aib = α-amino isobutyric acid, gly = glycine, his = histidine, leu = leucine, met = methionine, phe = phenylalanine, trp = tryptophan, tyr = tyrosine, val = valine. ^3^ The additional atom distances, which contribute to the remote [5 + 1]- or [5 + 2]-coordination of the Sn atoms in these compounds, are: Sn⋯O 3.12 Å in **11b**, Sn⋯O 3.14 Å in **11d**, Sn⋯O 3.00 and 3.13 Å in **11e**, Sn⋯N 2.79 Å in **11g**, Sn⋯O 2.80 and 2.85 Å in **11i** and Sn⋯O 2.82 Å in **11k**.

**Table 3:** Bond lengths (Å) of the Ge-coordination spheres of crystallographically characterized Ge-complexes of type ***15***. The bond labels correspond to Figure 12. The values for each column are rounded to the same number of decimal places.

	Ref.	*R*′ ^1^	Ge–N (a)	Ge–N (b)	Ge–O (c)	Ge–O (d)	Ge–O (e)	Ge–O (f)
**15a**	[106]	CH_2_CH_2_	2.08	2.11	1.88	1.88	1.89	1.77
**15b**	[107]	CH_2_CH_2_	2.09	2.10	1.89	1.88	1.90	1.76
**15c**	[108]	CH_2_CHMe^2^	2.09	2.10	1.88	1.87	1.89	1.78
**15d**	[109]	1,2-C_6_H_10_^3^	2.07	2.13	1.88	1.86	1.89	1.77
**15e** ^4^	[110]	CH_2_CH_2_	2.09	2.09	1.88	1.90	1.91	1.76
			2.07	2.11	1.87	1.88	1.91	1.77
**15f**	[108]	CH_2_CH_2_	2.09	2.11	1.89	1.88	1.89	1.75

^1^ The dangling ligand arms *R* are -CH_2_COOH (in **15a**, **15c**, **15d**), -CH_2_COO as *N*,*N*′-diphenylguanidinium salt (in **15b**), -CH_2_CH_2_OH (in **15e**) and a zwitterionic form of CH_2_CH_2_N(CH_2_COOH)_2_ (in **15f**). ^2^ The Me group of the backbone is bound to the C atom at the tripod-center N-donor atom. ^3^ *trans*-1,2-cyclohexanediyl backbone. ^4^ Data are listed for two crystallographically independent molecules in the asymmetric unit.

**Table 4:** Bond lengths (Å) of the Ge-coordination spheres of crystallographically characterized heteronuclear complexes of type ***17***. The bond labels correspond to Figure 12. The values for each column are rounded to the same number of decimal places.

	Ref.	*ML_n_*	Ge–N (a)	Ge–O (b)	Ge–O (b’)	Ge–O (c)	Ge–O (d)	Ge–O (e)
**17a**	[103]	La(H_2_O)_4_	2.10	1.93	1.93	1.86	1.81	1.80
**17b**	[113]	Nd(H_2_O)_4_	2.10	1.93	1.92	1.86	1.81	1.80
**17c**	[114]	Tb(H_2_O)_3_	2.10	1.93	1.92	1.87	1.81	1.81
**17d**	[115]	Tm(H_2_O)_3_	2.10	1.93	1.91	1.87	1.81	1.81
**17e**	[114]	Yb(H_2_O)_3_	2.10	1.93	1.92	1.86	1.81	1.81
**17f**	[116]	Cu(H_2_O)	2.09	1.91	1.91	1.84	1.90 ^1^	1.79

^1^ In compound **17f**, the ligand “O” is H_2_O.

**Table 5:** Bond lengths (Å) and C-Sn-C angles (deg.) of the Sn-coordination spheres of complexes **18a**–**18g**. The positions of *L*, *R* and *R*′ correspond to ***18*** in Figure 13. The values for each column are rounded to the same number of decimal places.

	Ref.	*L*,*R*,*R*′	C-Sn-C	Sn–N	Sn–O ^1^	Sn–O ^2^	Sn–O(*L*)	Sn⋯O
**18a**	[119]	-,H,Me	134.3	2.27	2.17	2.17	n/a	n/a
**18b**	[120]	-,*m*Tol,*n*Bu	132.4	2.23	2.13	2.14	n/a	n/a
**18c**	[121]	H_2_O,H,Me	161.1	2.29	2.20	2.37	2.38	2.79
**18d**	[122]	H_2_O,Me,Me	162.5	2.36	2.19	2.35	2.36	2.74
**18e**	[123]	MeOH,H,Me	159.5	2.28 ^3^	2.16	2.35	2.37	2.95
**18f**	[124]	H_2_O,H,*n*Bu	162.7	2.32	2.21	2.35	2.36	2.77
**18g**	[125]	H_2_O,H,*n*Bu	163.4	2.30	2.20	2.33	2.31	2.76

^1^ This bond is to the non-bridging carboxylate O of the (O,N,O)-ligand. ^2^ This bond is to the bridging carboxylate O of the (O,N,O)-ligand, which is involved in remote O⋯Sn-coordination with an adjacent complex molecule. ^3^ Average value from two disorder positions of the N atom site.

## Data Availability

Not applicable.

## Appendix A

**Table A1:** List of literature references (Ref.) and CSD reference codes (CSD Refcode) of crystallographically characterized compounds mentioned in this paper with Roman numerals. The crystallographic data (in CIF format) can be accessed from the Cambridge Structural Database (CSD) using the respective CSD Refcode via https://www.ccdc.cam.ac.uk/structures/ (accessed on 1 February 2025 for availability check).

Compound	Ref.	CSD Refcode	Compound	Ref.	CSD Refcode	Compound	Ref.	CSD Refcode
**I**	[26]	IHAFAY	**XIVe**	[151]	KAXVIP	**XXIII**	[167]	FEYMON
**II**	[27]	EYASOO	**XIVf**	[155]	OGOCIZ	**XXIVa**	[168]	RABLUB
**III**	[28]	MOCCIT	**XIVg**	[156]	NEYDED	**XXIVb**	[168]	RABMAI
**IV**	[29]	DMSNQN	**XIVh**	[157]	JATHUI	**XXV**	[169]	IJAXEW
**V**	[30]	FEDYOC	**XVa**	[152]	ZEHQIP	**XXVI**	[170]	OGABAD
**VI**	[31]	FICNUA	**XVb**	[153]	KALYEE	**XXVII**	[173]	QIFBIT
**VII**	[32]	CEWJAP	**XVc**	[154]	ESOMAC	**XXVIIIa**	[174]	UGEKOK
**VIII**	[33]	WONQOG	**XVI**	[158]	JILXAH	**XXVIIIb**	[174]	UGELEB
**IX**	[34]	CEKWAR	**XVII**	[159]	ZIGWIY	**XXVIIIc**	[174]	UGELAX
**X**	[35]	WEQRIU	**XVIII**	[159]	ZIGTUH	**XXVIIId**	[174]	UGELIF
**XI**	[36]	VUKMAS	**XIXa**	[161]	ACIJED	**XXVIIIe**	[174]	UGEKUQ
**XII**	[89]	VUNKAR	**XIXb**	[159]	ZIGWAG ^1^	**XXIX**	[179]	KISROT
**XIII**	[90]	IQATOK	**XXa**	[162]	MUFQAH	**XXX**	[180]	QIHWEJ
**XIVa**	[148]	JUNHAB	**XXb**	[163]	OFOYOZ	**XXXI**	[181]	JIPYEP ^2^
**XIVb**	[149]	TOZNEB	**XXIa**	[164]	JICTAU	**XXXII**	[184]	SUPHOC
**XIVc**	[150]	EHIZOK	**XXIb**	[165]	YILVOI	**XXXIII**	[185]	GIJJIT
**XIVd**	[151]	KAXVEL	**XXII**	[166]	WEKFAU	**XXXIV**	[185]	GIJJAL

^1^ Another entry of a crystal structure of this compound can be found under CSD Refcode ZIGWEU. ^2^ Another entry of a crystal structure of this compound can be found under CSD Refcode JIPYOZ.

**Table A2:** List of literature references (Ref.) and CSD reference codes (CSD Refcode) of crystallographically characterized compounds mentioned in this paper with Arabic numerals. The crystallographic data (in CIF format) can be accessed from the Cambridge Structural Database (CSD) using the respective CSD Refcode via https://www.ccdc.cam.ac.uk/structures/ (accessed on 1 February 2025 for availability check).

Compound	Ref.	CSD Refcode	Compound	Ref.	CSD Refcode	Compound	Ref.	CSD Refcode
**1**	[48]	WUYTIU	**11g**	[78]	KIHYAB	**17e**	[114]	GELJUE
**2a**	[48]	WUYTOA	**11h**	[79]	VUPRON	**17f**	[116]	RAGHIS
**2b**	[48]	WUYTOG	**11i**	[80]	IGUQIL	**18a**	[119]	OWEYAS
**2c**	[48]	WUYVAO	**11j**	[81]	MOCRAY	**18b**	[120]	IGEYOI
**2d**	[48]	WUYVES	**11k**	[81]	MOCREC	**18c**	[121]	KELLAO
**2e**	[48]	WUYVIW	**11l**	[82]	VUPDAL	**18d**	[122]	TOSRIC
**3a**	[49]	GAGJEE	**11m**	[83]	PATHEX	**18e**	[123]	GAMYUP
**3b**	[49]	GAGJII	**11n**	[83]	PATHIB	**18f**	[124]	SARREL
**3c**	[49]	GAGJOO	**11o**	[84]	SASZUH	**18g**	[125]	LUKDEB01
**3d**	[50]	JAZHAU	**11p**	[85]	GLDPSN	**19**	[126]	VAZHAG
**3e**	[50]	JAZHEY	**12a**	[93]	CASYOK	**20**	[127]	VIPDAZ
**3f**	[50]	JAZHOI	**12b**	[94]	CECXEN	**21**	[128]	QOVVEE
**5**	[52]	UYAYEZ	**12c**	[95]	FMESIA	**22**	[129]	QINWOZ
**6a**	[39]	JAPZOT	**12d**	[95]	FMESIB	**23**	[130]	HAWBOW
**6b**	[37]	HIVJOP	**12e**	[96]	FOFXED	**24**	[131]	LOFJEV
**6c**	[37]	HIVJUV	**12f**	[97]	GEKXID	**25** ^1^	[132]	FIBWAO
**7a**	[57]	GLYMSN10	**12g**	[98]	DIRSAY	**25** ^2^	[132]	FIBWOC
**7b**	[58]	BIGSUG	**13**	[102]	VUHCIK	**25** ^3^	[133]	IWEROR
**8a**	[59]	ECYSSN	**13′**	[103]	LAFHIL	**25** ^4^	[133]	IWERIL
**8b**	[60]	BONYAE	**14**	[104]	TEBJOA	**26** ^5^	[134]	HIVZUJ
**8c**	[61]	VUKLIW	**15a**	[106]	VEFGOC	**26** ^6^	[135]	SNEDTA
**9a**	[39]	JAPZUZ	**15b**	[107]	VOCPIN	**26** ^7^	[136]	NUQZEE
**9b**	[51]	QANSOR	**15c**	[108]	YUFYIH	**26** ^8^	[137]	QELZUC
**10a**	[70]	VASWER	**15d**	[109]	XOHHEH	**26** ^9^	[137]	QEMBAL
**10b**	[71]	VAWHEG	**15e**	[110]	VOCPOT	**26** ^10^	[138]	TORTUP
**11a**	[73]	DOHBIL	**15f**	[108]	YUFYON	**26** ^11^	[139]	WEVNAP
**11b**	[74]	NOCQOM	**16**	[111]	XUZZEZ	**27** ^12^	[140]	KIJWUV
**11c**	[75]	QOBXOU	**17a**	[103]	GEMPEV	**27** ^12^	[141]	KIJWUV01
**11d**	[76]	VUKHAK	**17b**	[113]	ELOYIO	**27** ^13^	[142]	LOHLEZ
**11e**	[76]	VUKHEO	**17c**	[114]	GELJOY	**28**	[143]	EDTASN
**11f**	[77]	ZUQBOC	**17d**	[115]	XAVRUL	**29**	[144]	HEDTAT

^1^ Potassium salt. ^2^ Rubidium salt. ^3^ Methylammonium salt. ^4^ Guanidinium salt. ^5^ *L* = HO(SnCl_3_OH_2_). ^6^ *L* = H_2_O. ^7^ *L* = OH^–^ (counter-ions: Ba^2+^, Cl^–^). ^8^ *L* = OH^–^ (counter-ion: Ba^2+^). ^9^ *L* = F^–^ (counter-ion: NH_4_^+^). ^10^ *L* = OH^–^ (counter-ion: Na^+^). ^11^ *L* = Cl^–^ (counter-ion: H_3_O^+^). ^12^ counter-ion: H_3_O^+^. ^13^ counter-ion: NH_4_^+^.

**Table A3:** List of literature references (Ref.) and CSD reference codes (CSD Refcode) of selected crystallographically characterized compounds mentioned in this paper. The crystallographic data (in CIF format) can be accessed from the Cambridge Structural Database (CSD) using the respective CSD Refcode via https://www.ccdc.cam.ac.uk/structures/ (accessed on 1 February 2025 for availability check).

Entry	Ref.	CSD Refcode	Entry	Ref.	CSD Refcode	Entry	Ref.	CSD Refcode
**A3a**	[66]	BOHNES	**A3h**	[122]	TOSRUO	**A3o**	[177]	PEGRUQ
**A3b**	[67]	ODEWEA	**A3i**	[146]	EDTAFE01	**A3p**	[178]	ZAYRIC
**A3c**	[68]	RIRRUG	**A3j**	[147]	CERFEK	**A3q**	[182]	TASSAH
**A3d**	[69]	NAGZAZ	**A3k**	[171]	TIRYAU	**A3r**	[183]	TUPMEW
**A3e**	[112]	DANRUG	**A3l**	[171]	TIRXUN	**A3s**	[186]	IGICEG
**A3f**	[117]	XAVROF	**A3m**	[172]	AMSOCU	**A3t**	[187]	RODLOL
**A3g**	[118]	TEKSEJ	**A3n**	[176]	JOVXAV			
